# Importin/exportin-mediated nucleocytoplasmic shuttling of cucumber mosaic virus 2b protein is required for 2b’s efficient suppression of RNA silencing

**DOI:** 10.1371/journal.ppat.1010267

**Published:** 2022-01-26

**Authors:** Hangil Kim, Hanako Shimura, Kae Sueda, Chikara Masuta

**Affiliations:** Research Faculty of Agriculture, Hokkaido University, Sapporo, Hokkaido, Japan; University of California, Davis Genome Center, UNITED STATES

## Abstract

The 2b protein (2b) of cucumber mosaic virus (CMV), an RNA-silencing suppressor (RSS), is a major pathogenicity determinant of CMV. 2b is localized in the nucleus and cytoplasm, and its nuclear import is determined by two nuclear localization signals (NLSs); a carrier protein (importin [IMPα]) is predicted to be involved in 2b’s nuclear transport. Cytoplasmic 2bs play a role in suppression of RNA silencing by binding to small RNAs and AGO proteins. A putative nuclear export signal (NES) motif was also found in 2b, but has not been proved to function. Here, we identified a leucine-rich motif in 2b’s C-terminal half as an NES. We then showed that NES-deficient 2b accumulated abundantly in the nucleus and lost its RSS activity, suggesting that 2b exported from the nucleus can play a role as an RSS. Although two serine residues (S40 and S42) were previously found to be phosphorylated, we also found that an additional phosphorylation site (S28) alone can affect 2b’s nuclear localization and RSS activity. Alanine substitution at S28 impaired the IMPα-mediated nuclear/nucleolar localization of 2b, and RSS activity was even stronger compared to wild-type 2b. In a subcellular fractionation assay, phosphorylated 2bs were detected in the nucleus, and comparison of the accumulation levels of nuclear phospho-2b between wild-type 2b and the NES mutant showed a greatly reduced level of the phosphorylated NES mutant in the nucleus, suggesting that 2bs are dephosphorylated in the nucleus and may be translocated to the cytoplasm in a nonphosphorylated form. These results suggest that 2b manipulates its nucleocytoplasmic transport as if it tracks down its targets, small RNAs and AGOs, in the RNA silencing pathway. We infer that 2b’s efficient RSS activity is maintained by a balance of phosphorylation and dephosphorylation, which are coupled to importin/exportin-mediated shuttling between the nucleus and cytoplasm.

## Author summary

RNA silencing, a small RNA (sRNA)-mediated sequence-specific mechanism, is a primary antiviral response of host plants against viral infection. Argonaute 1 (AGO1), an effector protein of RNA silencing, associates with sRNAs to degrade viral RNAs or silence the genes involved in biotic stresses in cytoplasm; such functions are promoted by its translocation between the nucleus and cytoplasm. To survive host RNA silencing, cucumber mosaic virus (CMV) encodes an RNA silencing suppressor (RSS), 2b protein (2b). 2b plays multiple roles in suppressing RNA silencing by binding to either sRNAs or AGOs, in the nucleus and the cytoplasm. However, several previous reports suggest that 2b exerts its RSS activity mainly when it is in the cytoplasm. Here, we found that the serine residue at position 28 is phosphorylated, and its phosphorylation negatively regulated the RSS activity of 2b by regulating 2b’s nuclear/nucleolar localization. Furthermore, we also found that 2bs were dephosphorylated in the nucleus and exported to the cytoplasm in an exportin-dependent manner. Because 2b’s transport between the nucleus and cytoplasm is similar to that of AGOs, 2b seems to work in cells as if it is manipulating its nucleocytoplasmic trafficking to track down its targets in the RNA silencing pathway.

## Introduction

The nuclear envelope is a physical barrier that regulates dynamic compartmentation of proteins between the nucleus and cytoplasm [1]. Because this compartmentation of proteins is critical for signal transduction and gene regulation in the context of cell proliferation, differentiation and transformation, nucleocytoplasmic partitioning is accomplished by highly selective processes [2]. Nuclear import of proteins that contain a nuclear localization signal (NLS) is generally mediated by the karyopherin family of proteins, the importins (IMPs) [3]. IMPs directly or indirectly recognize the NLS motif of the target protein to guide it into the nucleus [1]. Post-translational modifications such as phosphorylation and conformational changes of proteins can expose or conceal the NLS motif to promote or compromise nuclear localization of the modified proteins in both animal and plant cells [4–10].

In contrast, the export of a protein from the nucleus is generally mediated by nuclear export signals (NESs). NESs are mostly leucine-rich motifs that provide an epitope for the interaction with a transporter of nuclear export, exportin [11,12]. Much less is known about the nuclear export mechanism of proteins in plants compared with nuclear import. In plant proteins, the function of tomato heat stress transcription factor HsfA2 has been reported to be controlled by the balance of NLS and NES [13]. Similarly, several studies have shown the nuclear export of virus proteins and its regulation in plants. For example, the nuclear export of the P6 protein of a DNA virus, cauliflower mosaic virus (CaMV), is mediated by the leucine-rich motif in the N-terminus, which is recognized by an exportin, CRM1 known as XPO1 [14]. The tomato leaf curl Java virus (ToLCJAV) capsid protein also has an NES at the C-terminal region for its nuclear export [15]. Nucleocytoplasmic shuttling of the tomato leaf carl Yunnan geminivirus (TLCYnV) C4 protein is mediated by post-translational phosphorylation and myristoylation, and its interaction with XPO1 is required for its nuclear export [16]. For RNA viruses, the NES motif of beet necrotic yellow vein virus (BNYVV) protein p25 is involved in p25’s nuclear export [17].

Cucumber mosaic virus (CMV) has three positive-sense, single-stranded genomic RNAs (RNAs 1–3) [18]; it encodes five genes on the genomic RNAs and two subgenomic RNAs (RNA4 and RNA4A) [19,20]. In plants infected with CMV strains including CMV-Y, cyclic symptoms are often observed; leaves with high and low levels of viral accumulation alternate with severe and mild symptoms, respectively. The 2b protein (2b) is involved in symptom induction, viral movement and counter-actions against host RNA silencing and salicylic acid (SA)- and jasmonic acid (JA)-related defense responses [21–28]. 2b has potential phosphorylation sites for casein kinase II (CKII) (S/TxxD/E) and cyclin-dependent kinase 2 (40SP41) at residues 39–43 (KSPSE) [29,30], and phosphorylation at serine residues 40 and 42 has been proven [31].

CMV 2b, one of the most-studied RNA silencing suppressors (RSSs) in plant viruses, counteracts RNA-silencing-mediated host defense [32] by disturbing multiple steps in the host RNA silencing pathway, but the most critical property in the silencing suppression is its ability to bind small RNAs (sRNAs), thus sequestering them and preventing the formation of the RNA-induced silencing complex (RISC) [21]. CMV 2b directly binds small interfering RNA (siRNAs) or micro RNAs (miRNAs) *in vivo* or *in vitro*, neutralizing the siRNA- or miRNA-mediated post-transcriptional gene silencing (PTGS) [22,30], and it also binds 24-nt siRNAs disrupting transcriptional gene silencing (TGS) [33,34]. Based on a subcellular distribution analysis, the nucleus or cytoskeleton of host cells was shown to be enriched in 2b [35], but comparable levels were also found in the cytoplasm [36]. CMV 2b has two NLSs containing an arginine-rich domain at residues 22–27 and 33–36, which are recognized by *Arabidopsis* IMPα for import into the nucleus or nucleolus [27,37], and the motifs overlap with the domain responsible for sRNA-binding [30]. The difference in the accumulation of 2b between the nucleus and cytoplasm seems to be linked to 2b’s functions. In the nucleus/nucleolus, 2b causes accelerated symptom appearance, but cytoplasm-localized 2b has a primary role in suppressing RNA silencing [21,36].

The 2b protein also can interact with Argonautes (AGOs), a crucial component of the RNA silencing machinery [28,33,37] with a central role in the cleavage of target RNAs in the cytoplasm. More recently, however, AGO1 was reported to bind to chromatin in the nucleus and promote gene expression in response to hormones and stresses, a novel role for AGO1 [38]. AGO1 is actually a shuttle protein between the cytoplasm and the nucleus and has both NLS and NES amino acid sequences [39]. Bologna and colleagues proposed a revised model of the plant miRNA pathway in which mature miRNA translocation to the cytoplasm is mediated by its association with AGO1 and CRM1 [39]. Furthermore, phosphorylation of a tyrosine residue (Y529) in human AGO2 inhibits AGO binding to sRNAs [40]. Based on those studies on AGOs, we here reconsidered the cellular functions of 2b because AGO and 2b share some common features: They are both localized in the nucleus and cytoplasm, where they interact with each other, and both are phosphorylated. Because the plant ortholog of human AGO2 is reportedly AGO1 [41], we confirmed the amino acid sequence similarity of the two proteins by multiple sequence alignments, suggesting that they also are structurally similar. In addition, both 2b and AGO1 can bind sRNAs [21,22,42]. For 2b’s siRNA-binding ability, Nemes et al. (2017) previously predicted *in silico* that 2b’s siRNA-binding is affected by the phosphorylation status at certain amino acid residues (serine residues 40 and 42, S40 and S42) [31]. Now, some questions are raised. First, if the serine residue 28 (S28) in the immediate vicinity of the sRNA-binding motifs of 2b is phosphorylated, as in the case of Y529 in human AGO2, is the sRNA-binding activity of 2b also inhibited? Because when human AGO2 was phosphorylated near the RNA-binding site, it lost its sRNA-binding ability; and thus, 2b might also lose its sRNA-binding when phosphorylated. Second, considering that AGO1 is translocated from the nucleus to the cytoplasm, is 2b also exported from the nucleus to locate AGOs and sRNAs?

In previous studies, CMV 2b was reported to be localized in the nucleus and cytoplasm and its nuclear localization determined by two NLS motifs, which may interact with a carrier protein for nuclear import, IMPα [27,36]. However, there has been no experimental evidence on 2b’s NES, even though a putative NES was predicted [29]. The purpose of this study was to clarify whether 2b can shuttle between the nucleus and cytoplasm to maintain efficient suppression of host RNA silencing. The relationship between 2b’s phosphorylation and its RSS activity is also discussed in light of the fact that 2b is shuttled between the nucleus and cytoplasm.

## Results

### 2b has NLS and NES motifs for nucleocytoplasmic translocation

To search for NES motif(s) in 2b, we used the NetNES v1.1 online program and found an NES motif in the C-terminal half (S1A Fig). For the analysis, 2bs of three CMV subgroup I strains, CMV-Y (Y2b), CMV-CM95R (R2b) and CMV-HL (HL2b), and a chimeric 2b, Y/HL2b (chimeric 2b between CMV-Y and CMV-HL), were used [22,43,44,45]. As shown in S1B Fig, a leucine residue (L87) of HL2b, R2b and Y/HL2b has a significantly high NES score, and a motif of residues from L77 to L87 has high values of hidden Markov model (HMM), a tool for protein domain identification [46], indicating that the residues from L77 to L87 may function as an NES. Unexpectedly, only Y2b contains a serine (S77) instead of a leucine, and its NES score was not significantly high (S1B Fig).

**Fig 1:**
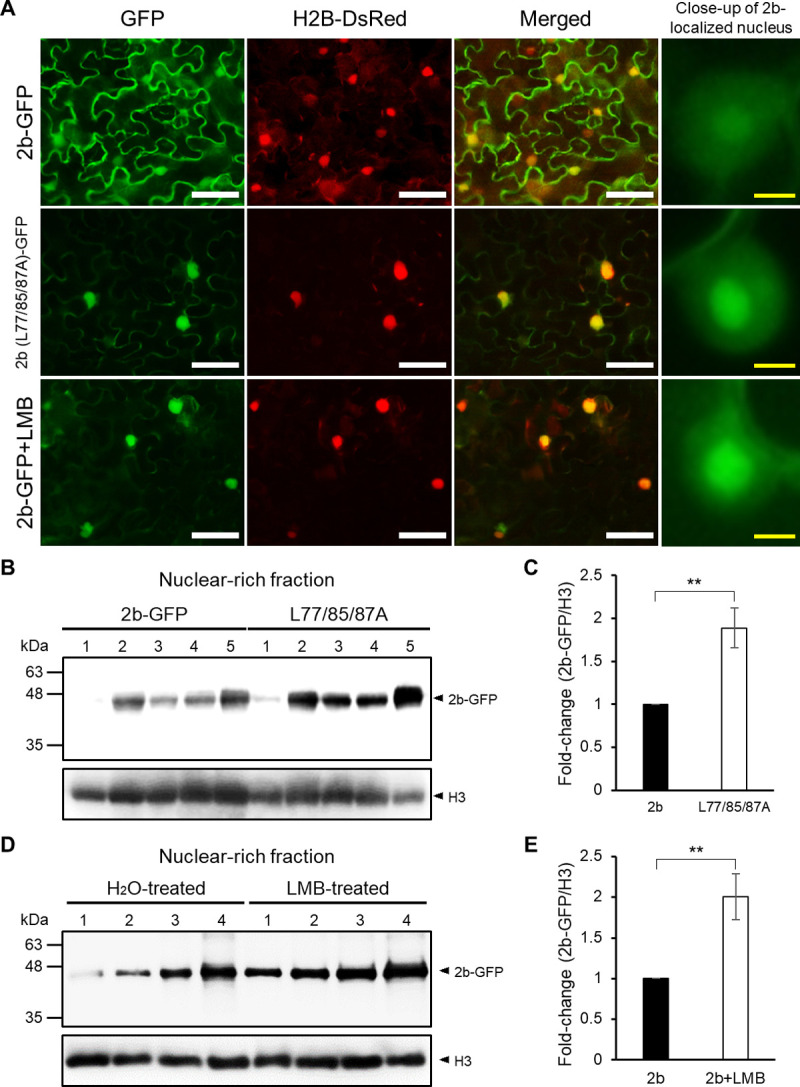
2b has an NES motif for translocation from nucleus to cytoplasm. (A-E) Effect of a mutation in NES on the subcellular localization of 2b in *N*. *benthamiana*. The GFP-tagged wild-type 2b (2b-GFP) or L77/85/87A was co-expressed with Ds-Red-fused histone 2B (H2B-DsRed), a nuclear marker protein, in *N*. *benthamiana* by agroinfiltration. LMB (40 nM) was infiltrated into the leaves at 2 days post agroinfiltration. (A) GFP fluorescence was observed with an epifluorescence microscope at 4 h after the LMB treatment. Scale bar: 50 μm (white), 5 μm (yellow). (B) Western blot of nuclear-rich fractions to detect 2b-GFP. The numbers above the blot are 5 replicates, and the identical numbers below 2b-GFP and L77/85/87A-GFP indicate the same leaves were used. The histone protein (H3) was detected using anti-H3 antibodies as a loading control. (C) Relative 2b-GFP/H3 ratios were densitometrically calculated using Multi Gauge Software (Fujifilm) and shown as a fold change. Mean values (±SE) were tested on log-transformed data for a significant difference using Student’s *t*-test (**P < 0.01). (D) Western blot analysis using the nuclear-rich fractions to detect 2b-GFP from the LMB-treated leaves. The identical numbers above the blot indicate the same leaves used. (E) Relative 2b-GFP/H3 ratios in the H_2_O-treated and LMB-treated samples were densitometrically calculated using Multi Gauge Software (Fujifilm) and shown as a fold change. Mean values (±SE) were tested on log-transformed data for a significant difference using Student’s *t*-test (**P < 0.01).

To verify the role of the predicted NES for 2b’s nuclear export, we generated a 2b mutant by substituting three leucine residues (L77, L85 and L87) with alanine residues (L77/85/87A) based on R2b of the CMV-CM95R strain because the leucine residues generally carry a role in NES by associating with AtXPO1 [12]. To investigate the effect of those mutations on 2b’s subcellular localization, we tagged the mutant 2bs with GFP, and these 2b-GFP constructs were transiently expressed in *Nicotiana benthamiana* leaves after insertion by agroinfiltration. As shown in Fig 1A, we found that accumulation of the mutant 2b was high in the nucleus and nucleolus compared to the amount of wild-type 2b. Those observations were further confirmed by western blot analysis using GFP antibodies and nuclear-rich fractions; the amount of mutant L77/85/87A in the nucleus was almost twice that of wild-type 2b (Fig 1B and 1C). We then used leptomycin B (LMB), an inactivator of XPO1 to test whether 2b’s localization was affected. LMB (40 nM) was infiltrated into the agroinfiltrated patches at 2 days post agroinfiltration, and the localization of 2b-GFP was observed at 4 h after the LMB treatment. 2b-GFP was found mainly in the nucleus, including the nucleolus, in the LMB-treated tissues, whereas 2b in the non-treated tissues accumulated in the cytoplasm and nucleus (Fig 1A). In addition, the 2b protein level in the nuclear-rich fraction was also significantly increased by the LMB treatment (Fig 1D and 1E). These results indicate the presence of NES in the C-terminal region from L77 to L87 and, thus, XPO1-mediated nuclear export.

We further investigated the nuclear export of Y2b into the cytoplasm because Y2b lacked an ideal NES when predicted by the NetNES v1.1 program. Because the CMV A1 vector has a truncated Y2b (residue 1 to 81, Y2bΔC) without NES, we examined whether the Y2b’s NES motif functions by cloning the DsRed gene into the A1 vector as a reporter for 2b accumulation (S2A Fig). As shown in S2B Fig, Y2b-DsRed was localized in both the nucleus and cytoplasm, whereas Y2bΔC-DsRed highly abundant in the nucleus and scarce in the cytoplasm, indicating that Y2b also contains a functional NES motif in the C-terminal region. By comparing Y2b and Y2bΔC, we revealed that the the C-terminal region including the NES motif was important for 2b’s nuclear localization. These results suggest that all subgroup I CMV 2bs have both NLS and NES motifs for nucleocytoplasmic translocation.

### Phosphorylation on S28 is required for 2b nuclear localization

When we scanned the NLS motifs of 2b, we noticed some serine residues near the NLSs, which might be phosphorylated. Because phosphorylation-mediated nuclear import is a common mechanism to localize proteins in the nucleus of eukaryotic cells [8], we expected that phosphorylation of the residues near the NLSs might affect 2b nuclear localization. To identify phosphorylation sites on 2b, we used the NetPhos v2.0 software (http://www.cbs.dtu.dk/services/NetPhos-2.0/) and found seven residues including S40 and S42 (S3A Fig), which were previously proven to be phosphorylated [31].

**Fig 2:**
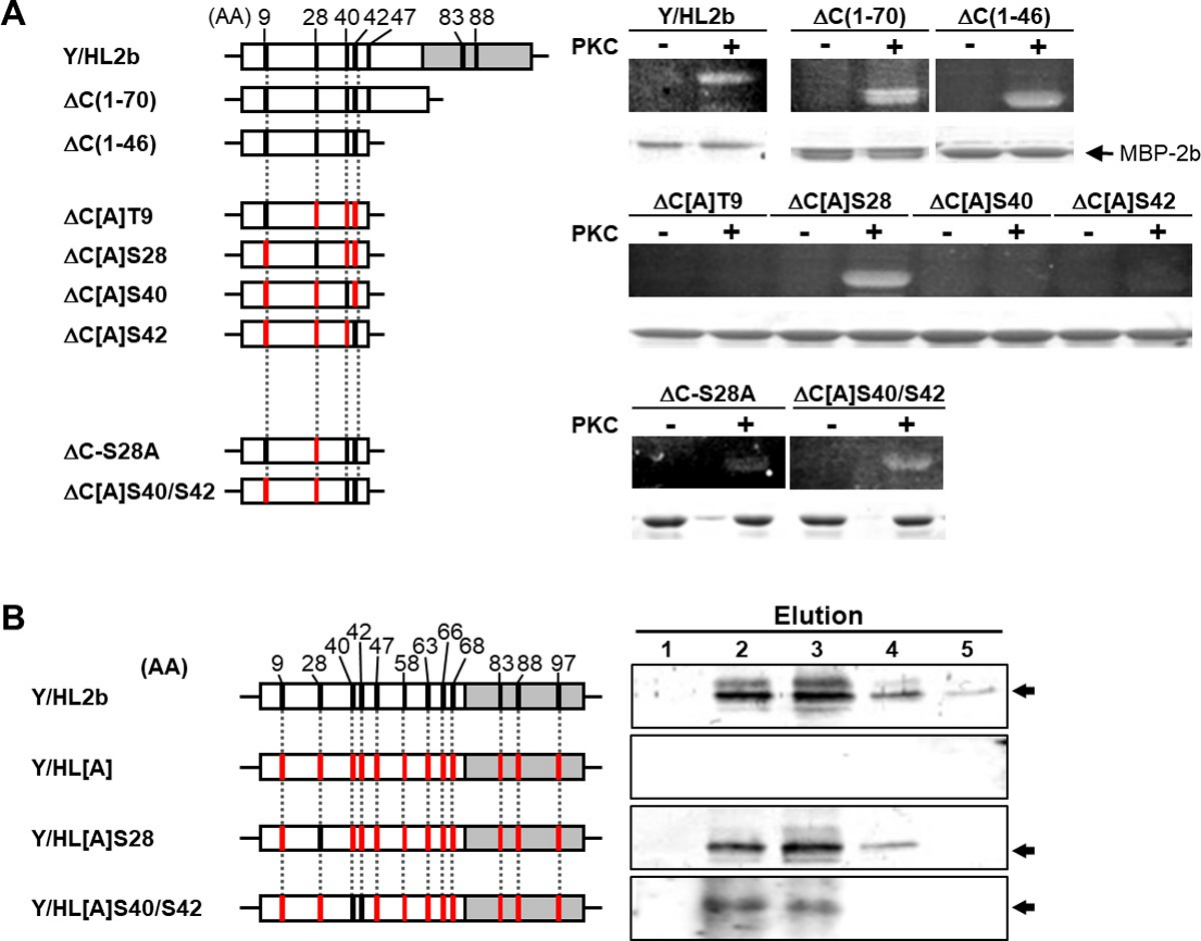
Mapping of phosphorylation sites of 2b. (A) Schematic structures of the 2b mutants expressed in *E*. *coli* and *in vitro* phosphorylation assay. White boxes: Y2b peptide sequence; gray boxes: HL2b peptide sequence. Black bars: serine (S) or threonine (T); red bars: alanine. When the mutant 2b backbone is the construct containing alanine-substituted residues on all the putative phosphorylation sites, the mutant names contain “[A]”. Purified mutant 2b proteins were co-incubated with (+) or without (-) PKC. Phosphorylated proteins were detected by phosphoprotein gel staining (upper panel); the same gel was stained with Coomassie brilliant blue (lower panel). (B) *In vivo* phosphorylation assay. White boxes: N-terminal half of Y2b; gray boxes: C-terminal half of HL2b. Black bars: putative phosphorylation residues (S, T or Y); red bars: alanine-substituted residues. Fraction numbers are above the lanes. Elution 1–5: Elution fraction 1–5. Arrows on right: phosphorylated 2bs.

To determine whether the predicted residues are phosphorylated, we assayed phosphorylation using 2b proteins with mutations at possible phosphorylation sites. For the phosphorylation assay, the 2b protein was produced in *Escherichia coli* as a C-terminal fusion with the maltose binding protein (MBP). We used a chimeric Y/HL2b protein (S3A Fig) because the E104 in the C-terminal region of Y2b is toxic to *E*. *coli* [47] (S1A Fig). *E*. *coli*-produced HL2b and Y/HL2b were treated with protein kinase C (PKC), separated by SDS-PAGE, and then the phosphorylated 2bs were visualized by phosphoprotein gel staining. As shown in S3B Fig, HL2b and Y/HL2b were clearly phosphorylated *in vitro*. For the *in vitro* phosphorylation using several truncated 2b constructs (Fig 2A), we found that the presence of the N-terminal half (46 amino acids) of 2b was sufficient for 2b phosphorylation.

To determine which amino acids (T9, S28, S40 and S42) were phosphorylated in this region, we created several alanine-substituted 2b mutants at the four sites. Among those mutants containing a single serine, only ΔC[A]S28 was phosphorylated by PKC (Fig 2A). Curiously, the mutants with only one serine at either S40 or S42 were not phosphorylated by PKC. On the other hand, the mutants containing both S40 and S42 were phosphorylated regardless of the alanine substitution at S28.

To confirm the phosphorylation of S28, S40 and S42 *in planta*, we examined *in vivo* phosphorylation of these serine residues. For the phosphorylation assay, we used the CMV-H1 RNA 2 vector in which the entire 2b open reading frame (ORF) was deleted and a multiple cloning site (MCS) was inserted; a stop codon for 2a was added before MCS (S4A Fig) [48]. The infection of the recombinant CMVs carrying wild-type 2b and 2b recombinants was confirmed by symptoms and western blot analysis (S4B and S4C Fig). Our western blot analysis revealed that anti-2b antibodies could efficiently detect the mutant 2bs as well as wild-type 2b, suggesting that the alanine substitution did not affect the antibody-binding to 2b (S4C Fig). Using a phospho-protein affinity column, we first isolated total phosphorylated proteins from tobacco tissues infected with CMV containing the 2b mutants (S5 Fig). Phosphorylated 2b was then detected using western blot analysis with anti-HL2b antibodies. As shown in Fig 2B, the positive control Y/HL2b was phosphorylated *in vivo*. We then prepared the 2b mutants containing an alanine at all the possible phosphorylation residues (Y/HL[A] in Fig 2B); in addition to the seven software-predicted amino acids, the other serine, threonine and tyrosine residues (Y58, S63, T66, S68 and T97) were all substituted with alanine. As expected, Y/HL[A] was not phosphorylated. On the other hand, Y/HL[A]S28 containing a serine at position 28 and Y/HL[A]S40/S42 containing a serine at positions 40 and 42 were phosphorylated *in planta*. These results clearly indicate that the three serine residues (S28, S40 and S42) are phosphorylated *in planta*.

**Fig 3:**
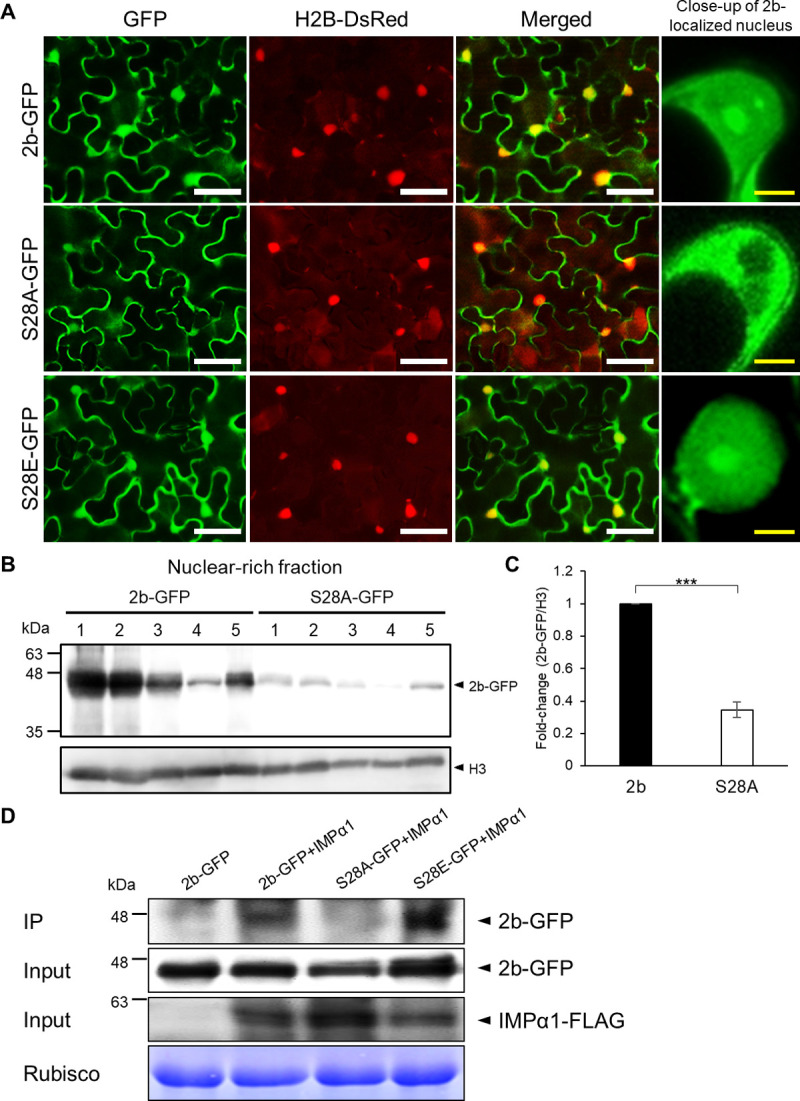
Effect of S28 phosphorylation on subcellular localization of 2b. (A) Subcellular localization of 2b and the 2b mutants in *N*. *benthamiana* cells. 2b-GFP and the GFP-tagged 2b mutants (S28A-GFP and S28E-GFP) were co-expressed with H2B-DsRed in agroinfiltrated leaves. GFP fluorescence was observed at 2 days post agroinfiltration using a confocal microscope. Scale bar: 50 μm (white) or 5 μm (yellow). (B) Detection of 2b and S28A in the nuclear-rich fraction. 2b-GFP and S28A-GFP were overexpressed in agroinfiltrated leaves, and the nuclear-rich fraction was probed for the proteins in a western blot. The GFP-tagged proteins and H3 (the loading control) were detected using anti-GFP antibodies and anti-H3 antibodies, respectively. The numbers above the blot are 5 replicate leaves; identical numbers indicate the same leaves were used for 2b-GFP and S28A-GFP. (C) Relative 2b accumulation levels. The relative 2b-GFP/H3 ratios were densitometrically calculated using Multi Gauge Software and shown as a fold-change. Mean values (±SE) were tested on log-transformed data for a significant difference using Student’s *t*-test (***P < 0.001). (D) *In vivo* co-immunoprecipitation of NbIMPα1 and 2b-GFP. NbIMPα1/2b-GFP complexes were first co-precipitated using anti-FLAG antibody, and 2b-GFP, S28A-GFP and S28E-GFP in the precipitant were detected by western blot analysis using anti-GFP antibodies. 2b-GFP, S28A-GFP, S28E-GFP and NbIMPα1-FLAG in the total protein extracts are shown as the inputs. RuBisCo large subunit is shown as a loading control.

Because S28 is located near the two NLSs (S3A Fig), we expected that nuclear localization of 2b might be affected by phosphorylation on S28. To determine the effect of S28 phosphorylation on 2b’s subcellular localization, the S28 residue was replaced with an alanine (S28A) to impair phosphorylation or with a glutamate (S28E) to mimic the state of phosphorylation, and then GFP was added to the C-terminus of 2b. The 2b-GFP sequences were cloned under the 35S promoter in a binary vector (pBE2113) and expressed in *N*. *benthamiana* by agroinfiltration. The wild-type 2b-GFP was detected in the nucleus and nucleolus as previously reported [37], and substantial levels were also found in the cytoplasm (Fig 3A). In contrast, the GFP signals of S28A were mainly localized in the cytoplasm and barely detected in the nucleus. Interestingly, even if the GFP signal was detected in the nucleus at a low level, it was never detected in the nucleolus (Fig 3A). When the phospho-mimetic mutant (S28E) was tested, GFP signals were found to be localized in the nucleus and cytoplasm, just like the wild-type 2b. In addition, when wild-type 2b and S28A were compared, 2b accumulation in the nuclear-rich fraction was dramatically reduced by the S28A substitution (Fig 3B and 3C). These results suggest that S28 is important for the 2b’s nuclear localization. We further analyzed the subcellular localization of S28E in two experiments using a NES mutant and with the exportin inhibitor LMB. As expected, both experiments confirmed that the impairment of the exportin-mediated 2b translocation resulted in an increase in nuclear accumulation of 2b (S6 Fig).

Wang et al. (2004) previously demonstrated that AtIMPα binds to the NLSs of 2b for nuclear transport [27] and that 2b enters the nucleus, perhaps in an AtIMPα-dependent manner. Because the S28 residue is just adjacent to the two NLSs, and the results of our subcellular localization study indicated that phosphorylation of S28 is required for 2b’s nuclear localization, we considered that phosphorylation of S28 was important for the 2b–IMPα interaction. Because the IMPα-mediated nuclear import of 2b had not been unequivocably proven, we first analyzed the involvement of IMPα in nuclear localization of 2b by silencing two AtIMPα homologs in *N*. *benthamiana* (NbIMPα1 and NbIMPα2), which share ~80% amino acid sequence identity with AtIMPα (S7A Fig). By virus-induced gene silencing (VIGS) using the PVX vector, both IMP genes were silenced by coinoculation with PVX-NbIMPα1 and PVX-NbIMPα2 (S7B and S7C Fig), while their viral RNA levels were not significantly different (S7D Fig). To examine whether IMPα-silencing affects nuclear localization of 2b, we used agroinfiltration to transiently express 2b-GFP in the IMPα-silenced *N*. *benthamiana* plants. As a result, 2b-GFP was clearly detected in both the nucleus and cytoplasm in the PVX vector-infected plants, and 2b nuclear localization was reduced in the IMPα-silenced plants (S8A Fig). Consistent with the microscopic observation, the level of 2b-GFP in the nuclear-rich fraction was slightly reduced (~12% reduction) in the IMPα-silenced *N*. *benthamiana* compared to that in the PVX-infected control plants (S8B and S8C Fig). When the free GFP was examined as a control, its nuclear localization was not affected by silencing the *IMPα* genes (S8D–S8F Fig). We anticipated that the 12% reduction in 2b nuclear localization was due to the recovery of IMPα expression by 2b’s RSS activity in the IMPα-silenced plants. To reduce 2b’s RSS activity, which may affect IMPα silencing, we then used the 2b mutant R46C, which lacks RSS activity and is localized in both the nucleus and cytoplasm [22] in the IMPα-silenced plants. As expected, the level of nuclear-localizing R46C-GFP was greatly decreased (~73% reduction) by the IMPα-silencing (S8G–S8I Fig). These data together suggest that the nuclear import of 2b is mediated by NbIMPα.

To determine whether the phosphorylation of S28 affects the interaction between 2b and NbIMPα, we performed a co-immunoprecipitation assay using NbIMPα1-FLAG and GFP-tagged 2bs (2b-GFP). The NbIMPα1-FLAG/2b-GFP complexes were first precipitated using anti-FLAG immunoglobulin G (IgG), then the 2b-GFP precipitants were detected by western blot analysis using anti-GFP IgG. As shown in Fig 3D, the wild-type 2b-GFP protein co-precipitated with NbIMPα1, indicating that 2b directly interacts with NbIMPα1. While S28A was not detected in the precipitants, the S28 phospho-mimetic mutant (S28E) was clearly detected. These results together suggest that phosphorylation at S28 is important for the 2b–NbIMPα1 interaction and thus 2b nuclear localization.

### Phosphorylation of 2b negatively affects RSS activity

**Fig 4:**
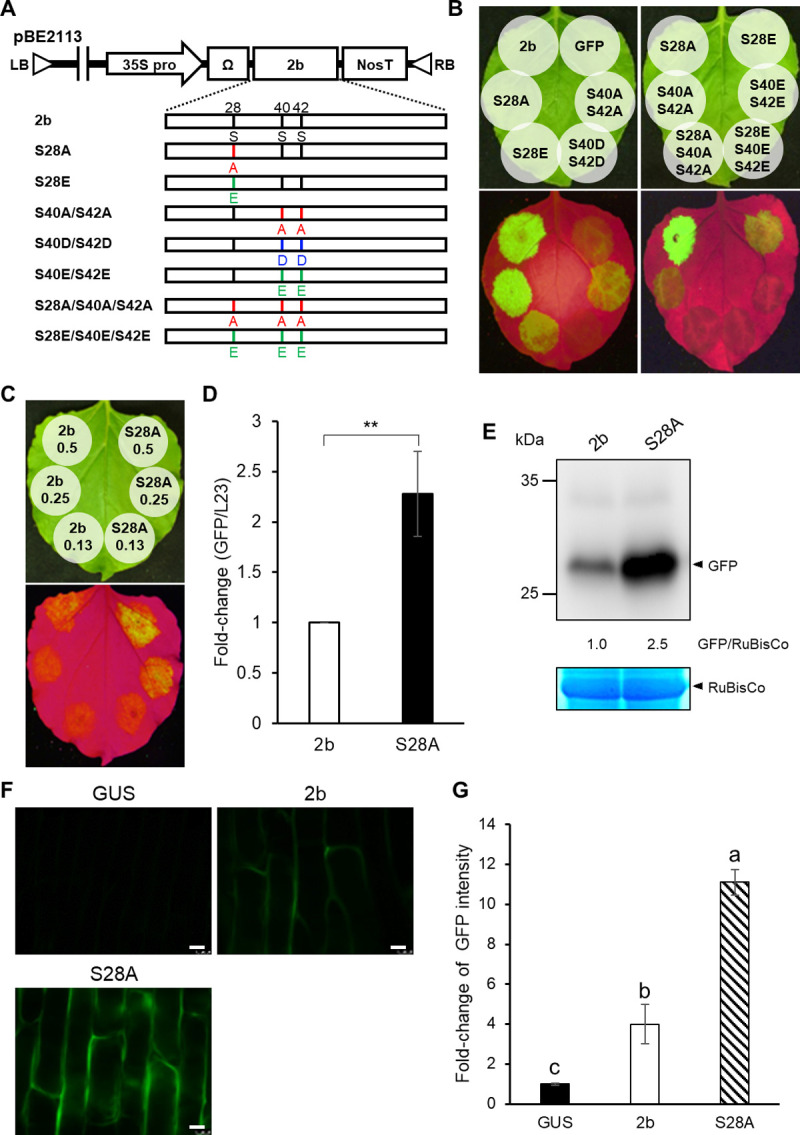
RSS activity of 2bs with mutations at phosphorylation sites. (A) Schematic structure of expression vector constructs containing mutant 2bs. The mutant 2bs were created by substituting S28, S40 and S42 with alanine (A), glutamic acid (E) or aspartic acid (D). (B) RSS activity of the mutant 2bs in the agroinfiltration transient expression assay. *Agrobacterium* cultures carried a 5:1:5 ratio of the GFP-sense (GFP), GFP-inverted-repeat (GFP-IR), and the 2b constructs. *N*. *benthamiana* leaves were agroinfiltrated with bacterial suspensions (~1.0 OD_600_). GFP was examined at 2 days post-infiltration. Inoculum containing GFP and GFP-IR was used as a control for GFP silencing. (C–E) Comparison of RSS activity between 2b and S28A in *N*. *benthamiana*. The *Agrobacterium* suspension of 2b or S28A was serially diluted to OD_600_ = 0.5, 0.25 and 0.13. The inocula were a mixture of *Agrobacterium* cultures carrying the GFP, GFP-IR and the 2b constructs. (C) Leaves were examined for GFP fluorescence at 2 days post-infiltration. (D) Real-time RT-PCR to compare GFP mRNA levels between 2b and S28A. The agroinfiltrated patches, where GFP, GFP-IR and 2b were simultaneously expressed, were excised. Mean values (±SE) are fold-changes when the value in 2b was set to 1.0. The values were analyzed on log-transformed data by Student’s *t*-test (**P < 0.01). (E) GFP in agroinfiltrated patches was detected by western blot analysis using anti-GFP antibodies. RuBisCo large subunit is shown as a loading control. The ratios of GFP/RuBisCo were densitometrically calculated when the value in 2b was set to 1.0. (F and G) Comparison of RSS activity between 2b and S28A in onion epidermal tissues. Onion bulb scales were infiltrated with a mixture of *Agrobacterium* suspension of GFP, GFP-IR and 2b. The plant experimental vector pBE2113 (GUS) was used as a negative control. GFP florescence was observed with an epifluorescence microscope. GFP fluorescence intensity was measured for three different onion scales using the LAS AF program (Leica). Scale bar: 50 μm. The values of GFP florescence are fold-changes when the value in GUS was set to 1.0. Means (±SE) were tested on log-transformed data for a significant difference using Tukey’s multiple comparison test (*P* < 0.05); different letters above the bars indicate a significant difference.

To analyze the relationship between the phosphorylation of 2b and its RSS activity, we generated a series of 2b mutants (alanine substitution at the phosphorylation sites and glutamate/aspartate substitution to mimic phosphoserine residues). We then examined the RSS activities of these 2b mutants in a conventional agroinfiltration assay in *N*. *benthamiana*. The 2b mutants were cloned into pBE2113 (Fig 4A), and RNA silencing of the GFP gene was induced by an inverted repeat construct of GFP (GFP-IR). Silencing of the GFP gene was efficiently suppressed by 2b and S28A; S28A showed even stronger RSS activity compared to wild-type 2b (Fig 4B). On the other hand, S40A/S42A and S28E had clearly weaker RSS activity, while S28A/S40A/S42A, S40D/S42D, S40E/S42E and S28E/S40E/S42E had no RSS activity. Because the sRNA-binding ability of 2b is important for suppressing RNA silencing [21,22], we tried to elucidate whether the phosphorylation of 2b affects its binding with sRNAs by examining the sRNA-binding ability of 2b *in vitro* using the phospho-mimetic 2b mutant (S28E/S40E/S42E) (S9 Fig). The MBP-fused 2b and the phospho-mimetic 2b mutant were synthesized in *E*. *coli*, and the purified MBP-2bs were incubated with biotinylated 21-nt siRNAs. The 2b–siRNA complexes were co-precipitated with streptavidin-coupled beads. The precipitated 2bs were separated by SDS-PAGE and visualized by Coomassie brilliant blue (CBB) staining. As a result, wild-type 2b was co-precipitated with biotinylated siRNAs, whereas S28E/S40E/S42E mutant was barely detectable in the gel (S9 Fig). These results suggest that phosphorylation of the three serine residues (S28, S40 and S42) negatively affects 2b’s RSS activity by interfering with the 2b–siRNA interaction. To estimate how this phosphorylation would affect siRNA-binding in the 2b’s conformation, we next analyzed the tertiary structures of the 2b mutants using homology modeling. As shown in S10A Fig, we noted that a hydrogen (H)-bond was formed between S42 and the backbone phosphate of siRNA in the nonphosphorylated 2b. All the mutant models were consistent with our experimental observations on 2b’s RSS activity (Fig 4B). The H-bond disappeared after phosphorylation or glutamate/aspartate substitution of S42 (S10A Fig). We further calculated the distance between S42 and cytosine 14 of siRNA in the 2b-siRNA binding models. All the 2b mutants without RSS activity are located farther from the siRNAs (S10B Fig) than all the 2b mutants with RSS activity. We thus consider that this H-bond formation at S42 somehow contributes to 2b’s siRNA binding. On the other hand, in a previous report of Chen et al. (2008) [49], R28 of tomato aspermy virus (TAV) was predicted to form H-bond with siRNA. Because the R28 of TAV 2b is corresponding to S28 of CMV 2b and it is located inside of RNA binding motifs, we expected that the phosphorylation of S28 may also affect 2b’s RNA-binding ability. To determine whether the S28 phosphorylation affects 2b’s sRNA-binding, another *in vitro* sRNA-binding assay was performed. As shown in S11A Fig, the S28E substitution dramatically reduced 2b’s sRNA-binding ability suggesting that the S28 phosphorylation also negatively affect 2b’s RSS activity by disturbing sRNA-binding.

According to a previous study by Du et al. (2014), the cytoplasmic 2b is important for 2b’s RSS activity [36]. Because the S28A mutant, which lacks phosphorylation at S28, was primarily localized in the cytoplasm (Fig 3), we anticipated that the S28A mutant might have even stronger RSS activity than wild-type 2b. To compare RSS activity between 2b and S28A, we performed a comparative study on RSS activity by diluting the *Agrobacterium* inoculum. *Agrobacterium* suspensions containing either wild-type 2b or S28A construct was serially diluted and infiltrated together with GFP- and GFP-IR-expressing bacterial inocula. As expected, the GFP signal was indeed stronger in the tissues infiltrated with S28A compared to those with wild-type 2b; S28A was ~2.5 times higher (Fig 4C–4E).

To confirm the difference in GFP fluorescence between 2b and S28A, we then used onion epidermal tissues where GFP fluorescence is more easily observed than in *N*. *benthamiana*. As shown in Fig 4F and 4G, the GFP signal was ~2.5 times higher in the tissues with S28A than in those with wild-type 2b. These results therefore suggest that the S28 phosphorylation provides some negative effect on RSS activity by enhancing 2b’s nuclear localization and/or structurally inhibiting sRNA-binding.

**Fig 5:**
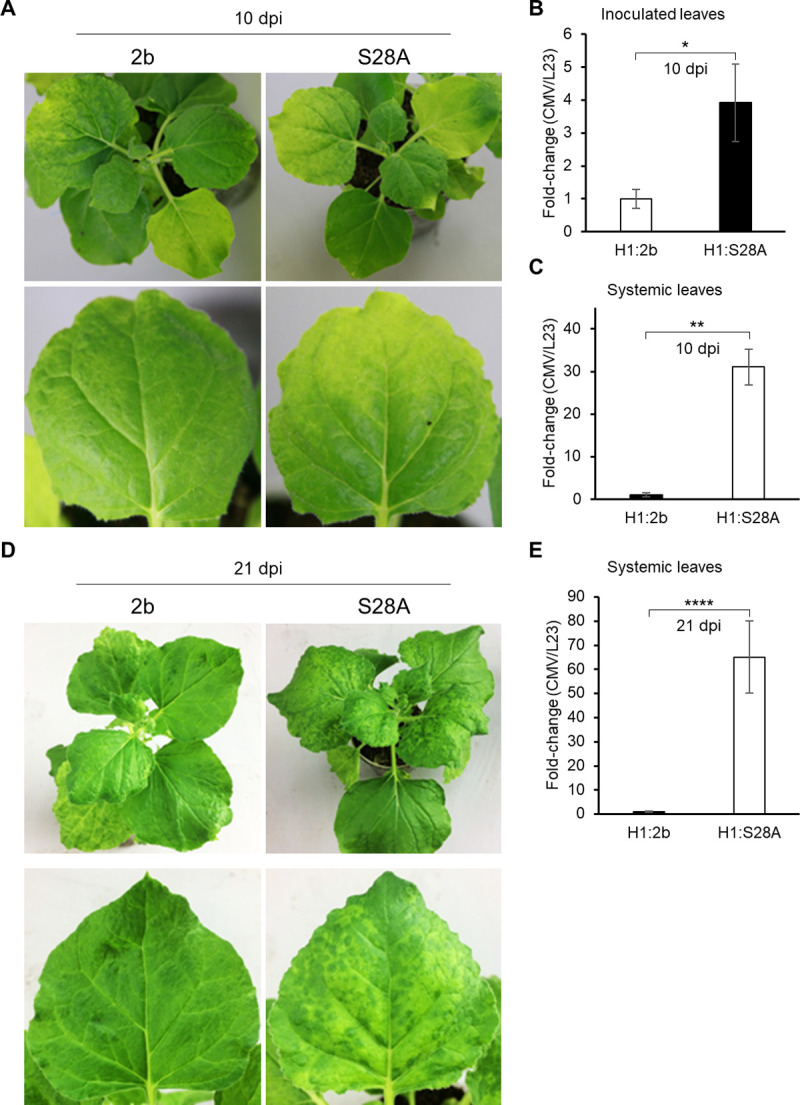
Effect of alanine substitution at S28 on CMV disease severity. (A) CMV symptoms on *N*. *benthamiana* 10 days post inoculation (dpi) with CMV containing S28A at early stage of infection. Wild-type 2b and S28A genes were inserted into the CMV-H1 vector (H1:2b and H1:S28A). CMV RNA levels based on real-time RT-PCR in inoculated (B) and systemically infected (Systemic) leaves (C). Means (±SE) are fold-changes, calculated when the value of H1:2b is set to 1.0, and tested on log-transformed data for a significant difference using Student’s *t*-test (*P < 0.05 or **P < 0.01). (D) Disease severity induced by 2b and 2b-S28A and (E) CMV RNA levels based on real-time qRT-PCR in systemically infected upper leaves at 21 dpi. Mean values (±SE) are fold-changes and statistically analyzed as above (****P < 0.0001).

Because S28A has stronger RSS activity than wild-type 2b (Fig 4), we considered that S28A might contribute to an increase in CMV accumulation by strongly suppressing antiviral RNA silencing. A recombinant CMV RNA 2 containing S28A (H1:S28A) was constructed and used with RNAs 1 and 3 of CMV-Y to co-inoculate *N*. *benthamiana*. The plants infected with CMV expressing S28A developed a systemic mosaic at 10 days post inoculation (dpi), earlier than those infected with CMV containing wild-type 2b (H1:2b), and the viral RNA level was ~4 times higher in the inoculated leaves and ~30 times higher in the upper systemic leaves than in the controls (Fig 5A–5C). Furthermore, compared to H1:2b, H1:S28A caused a very distinct yellowish mosaic in systemically infected leaves at 21 dpi, with ~65 times higher viral RNA level (Fig 5D and 5E). These data thus suggest that phosphorylation at S28 may reduce the level of 2b in the cytoplasm, and viral accumulation is thus lower due to the weaker RSS activity. To exclude the effect of other putative phosphorylation sites on viral disease severity, we used the 2b backbone, which includes alanine residues on all the putative phosphorylation sites except S28, S40 or S42, and three mutant 2bs ([A]S28, [A]S40/S42 and [A]S28/S40/S42) to test their ability to induce symptoms (S12A Fig). *N*. *benthamiana* plants inoculated with the CMV-H1 vector expressing [A]S28 were asymptomatic at 11 dpi, and the viral RNA level was ~20% of the level in plants infected with CMV-[A]S28/S40/S42 (S12B and S12C Fig). The plants infected with CMV expressing [A]S28/S40/S42 had relatively mild symptoms and an early symptom recovery at 11 dpi with a lower viral RNA level compared to the plants infected with CMV expressing [A]S40/S42 (S12B and S12D Fig). At 21 dpi, the plants infected with CMV-[A]S40/S42 had distinct mosaics, earlier than the plants with CMV-[A]S28/S40/S42, and the viral titer was significantly higher in the systemically infected leaves (S12E and S12F Fig), suggesting that S28A can enhance not only viral accumulation but also disease severity.

### NES-mediated nuclear export of 2b promotes 2b’s RSS activity

**Fig 6:**
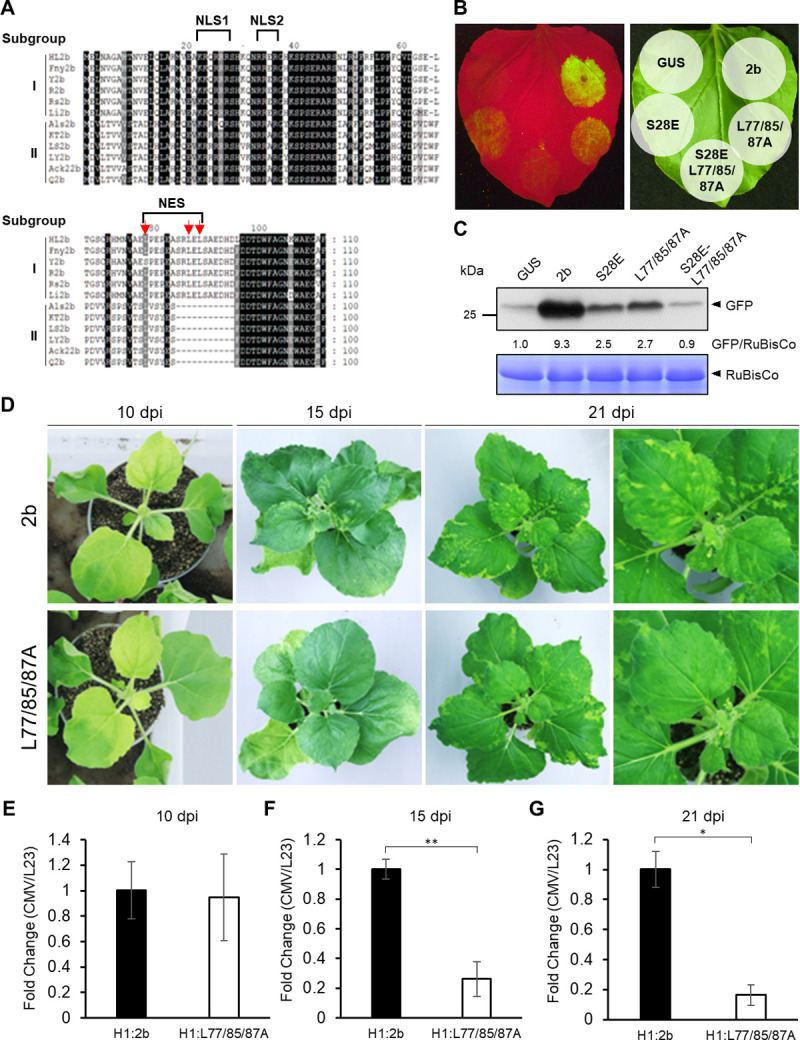
NES motif determines 2b’s RSS activity and CMV accumulation. (A) Alignment of 2b amino acid sequences belonging to subgroup I and II. Amino acid positions are indicated above the alignment. Red arrows indicate leucine residues important for 2b nuclear export. (B) RSS activity in 2b and NES mutant after infiltration of *N*. *benthamiana* leaves with *Agrobacterium* suspensions carrying GFP, GFP-IR and 2b at a ratio of 5:1:5. GFP florescence was observed at 2 days post-infiltration. (C) Western blot analysis to compare the GFP levels. GFP in the agroinfiltrated patches was detected by western blot analysis using anti-GFP antibodies. RuBisCo large subunit is shown as a loading control. The ratios of GFP/RuBisCo were densitometrically calculated when the value in GUS was set to 1.0. (D–G) Effect of a mutation in the NES motif on (D) CMV cyclic symptoms and (E–G) viral accumulation at 10, 15 and 21 dpi. *N*. *benthamiana* plants were inoculated with CMV containing either the wild-type 2b or the NES mutant (L77/85/87A). Viral RNA accumulation levels were detected by real-time RT-PCR. Means (±SE) are fold-changes calculated when the value in H1:2b was set to 1.0 and were tested on log-transformed data for a significant difference using Student’s *t*-test (**P < 0.01, *P < 0.05).

When we aligned the amino acid sequences of the 2bs of several CMV strains, we noticed that all of the 2b proteins from CMV subgroup II strains lacked the corresponding NES motif (Fig 6A), that is characteristic of all subgroup I strains. Because the 2bs of subgroup II had less efficient RSS activities [18,28], we further examined the effect of the mutations at NES on viral pathogenicity to determine whether nuclear accumulation of 2b indeed reduces 2b’s RSS activity. Three leucine residues in NES (L77, L85 and L87) were substituted with alanines in the backbone of R2b and its S28E mutant (L77/85/87A and S28E-L77/85/87A, respectively), which localized in the nucleus and the cytoplasm (Figs 1 and 3). Wild-type 2b strongly suppressed GFP gene silencing while S28E showed low RSS activity (Fig 6B and 6C). Unexpectedly, the RSS activity of 2b(L77/85/87A) was greatly reduced, and S28E-L77/85/87A were totally impaired, indicating that 2b’s RSS activity might be enhanced by the export of 2b to the cytoplasm (Fig 6B and 6C). In addition, our sRNA-binding assay revealed that the amino acid substitution in the NES site did not affect 2b’s RNA-binding ability (S11B Fig).

Although the RSS activity of 2b(L77/85/87A) was very low, it has been previously reported that a C-terminal deletion mutant without NES had RSS activity comparable to wild-type 2b [37]. To elucidate this discrepancy between the two observations, we here investigated the RSS activity of a series of C-terminal deletion mutants (S13A Fig). As the C-terminal amino acids were gradually trimmed on the background of 2b(L77/85/87A), the RSS activity suddenly recovered to a level similar to that of wild-type 2b between the 23-AA deletion [L77/85/87A(1–87)] and the 10-AA deletion [L77/85/87A(1–100)]. This result suggests that the 23 AA residues downstream of the NES site would affect the 2b’s RSS activity. Our western blot analysis showed that all the C-terminal deletion mutants were stably expressed in the agroinfiltrated tissues (S13C Fig).

To determine whether a lack of NES affects viral pathogenicity, we compared the disease severity between CMV with the intact 2b (H1:2b) and CMV with L77/85/87A (H1:L77/85/87A). At an early stage of infection (10 dpi), symptom severity and viral accumulation were not significantly different between H1:2b and H1:L77/85/87A (Fig 6D and 6E), but at 15 and 21 dpi, H1:2b had developed a systemic cyclic symptom on the upper leaves with a higher level of viral RNA accumulation (Fig 6D, 6F and 6G), unlike plants with H1:L77/85/87A. These results suggest that the nuclear export of 2b is necessary for efficient suppression of host antiviral RNA silencing.

### 2b is dephosphorylated in the nucleus

**Fig 7:**
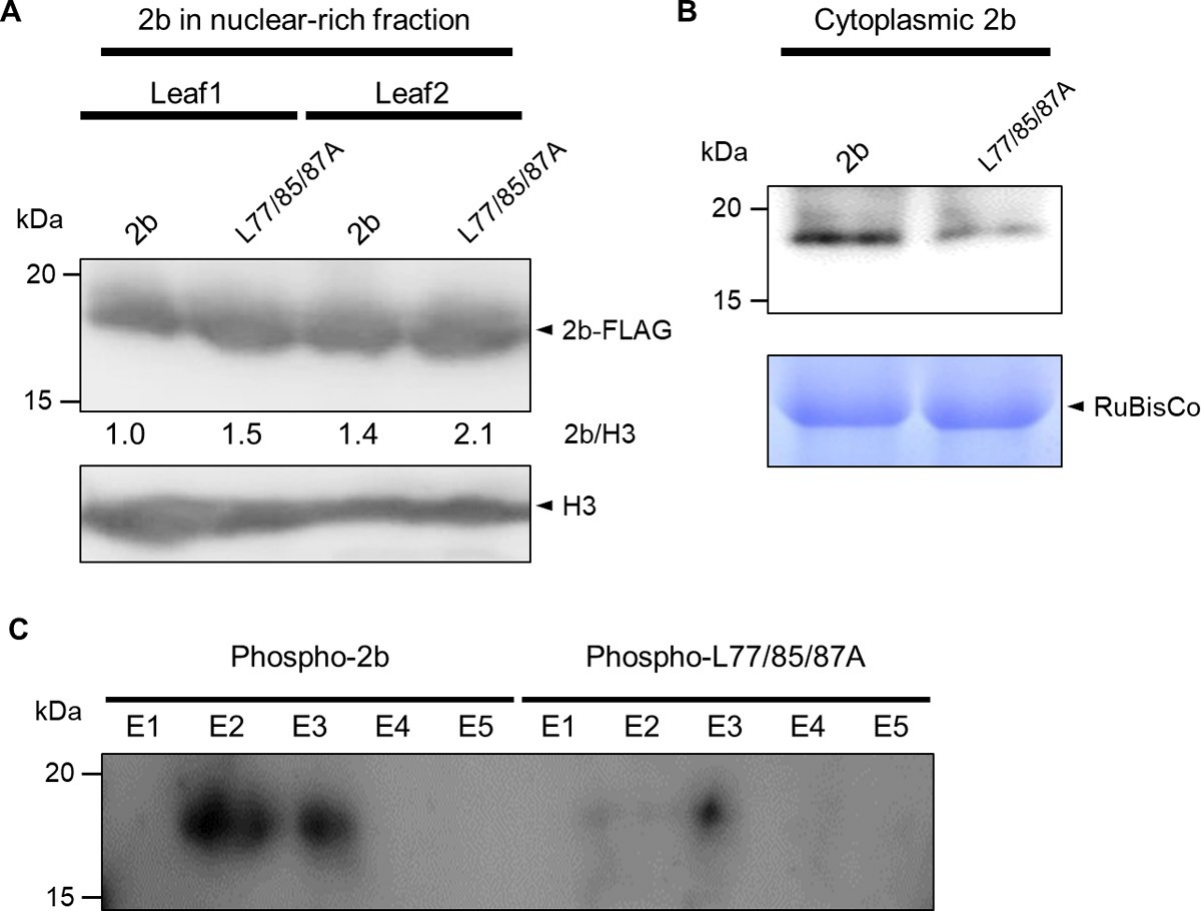
Comparison of levels of phosphorylated 2b in nucleus. (A) Western blot analysis to detect nuclear-localized 2b-FLAG protein in the nuclear-rich fraction from leaves of *N*. *benthamiana* expressing the FLAG-tagged 2b and L77/85/87A. Histone H3 was detected using anti-H3 antibodies for a loading control. Values below the blot for 2b are the relative ratio of 2b/H3. (B) Detection of cytoplasmic 2b by immunoprecipitation. The cytoplasmic fractions were separated from the total plant extracts by differentiated centrifugation. The 2b-FLAG was immunoprecipitated with anti-2b antibodies and detected in the western blot using anti-FLAG antibody. (C) Detection of phosphorylated 2b in the nuclear fraction. The level of phosphorylated 2b-FLAG in the nuclear-rich fraction was isolated using a phospho-protein enrichment column. Phosphorylated 2b in each elution (E1–5) was detected by western blot using anti-FLAG antibody.

Although S28A was not efficiently transported into the nucleus, it had very strong RSS activity. On the other hand, S28E was abundant in the nucleus, and S28E-L77/85/87A was also present in the nucleolus (S6 Fig). We thus wondered which form will predominate in the nucleus, the phosphorylated or nonphosphorylated 2b. To start to answer this question, we compared the accumulation levels of phosphorylated 2b in the nucleus with those of wild-type 2b and L77/85/87A. To isolate 2b from the nucleus, FLAG-tagged 2b and L77/85/87A were expressed in *N*. *benthamiana* leaves by agroinfiltration, and the nuclear fractions were isolated by sucrose density centrifugation. As expected from the results shown in Fig 1, the level of L77/85/87A-FLAG was higher than 2b-FLAG in the nucleus (Fig 7A). When we analyzed 2b isolated from the cytoplasmic fraction (S14 Fig), wild-type 2b was more abundant than L77/85/87A (Fig 7B). Then, we purified the phosphorylated 2b in the nuclear-rich fraction using a phosphoprotein-specific column (S5 Fig). In this experiment, we fractionated nuclei from the cells of agrobacterium-infiltrated tissues and tried to recover total protein containing 2b, but the recovery efficiency of 2b was not good enough to clearly detect phospho-2b by western blot using phosphoprotein staining. We thus decided to enrich phosphorylated proteins first by column chromatography and detect 2b in the eluted fractions using anti-FLAG antibody. As shown in the review by Schmidt et al. (2007) [50], the separation and enrichment of phosphoproteins using columns has been well studied, and excellent kits are now commercially available. The purified phospho-2bs were then detected by western blot analysis. As a result, we found that the phosphorylated 2b was indeed much more abundant than the phosphorylated L77/85/87A in the nucleus (Fig 7C). Considering that the nuclear export of L77/85/87A was impaired, we infer that phospho-2bs are likely dephosphorylated in the nucleus.

## Discussion

### Phosphorylation at S28 regulates 2b’s nuclear transport and RSS activity

In this study, we demonstrated that phosphorylation of S28 alone can determine 2b’s interaction with NbIMPα1 and its nuclear localization (Fig 3). According to Nardozzi et al. (2010), phosphorylation can either activate or inactivate IMP-mediated nuclear import [8]. For example, SV40 T-antigen has phosphorylation sites adjacent to the NLS, and the phosphorylation of SV40 T-antigen enhances NLS recognition by IMPα [2,51,52]. Because S28 is close to the two NLSs of 2b but not inside the NLS motifs, we hypothesized that 2b’s nuclear transport may be mediated by IMPα1 in the same fashion as that of SV40 T-antigen.

We here found that 2b could shuttle between nucleus and cytoplasm by the importin- and exportin-mediated transport systems. We also found that the phosphorylation of S28 was important for nuclear translocation. However, we consider that the role of the other phosphorylation sites (especially S40 and S42) in 2b’s subcellular localization is also important, and that combination of the phosphorylation of S28 and that of the other sites might determine the balance between the importin-mediated nuclear import and exportin-mediated nuclear export. For example, Neme et al. (2017) previously reported that S40D/S42D was localized to the cytoplasm [31]. On the other hand, because we here observed that S28A was localized to the cytoplasm, it is unclear whether the localization of 2b to the cytoplasm is due to the fact that S28 is not phosphorylated, or whether it is due to the fact that S40 and S42 are phosphorylated. One explanation for this is the balance between the 2b’s affinities for importin and exportin depending on the combination of phosphorylation at the three sites. If S28 is not phosphorylated, 2b cannot be transported to the nucleus by importin and thus localizes to the cytoplasm. On the other hand, S40D/S42D, in which S28 can be phosphorylated, will be transported to the nucleus by importin and then may be quickly exported from the nucleus by exportin. In other words, the phosphorylation state of 2b inside of the nucleus will be also important for exportin-mediated nuclear export. The relationship between the combination of phosphorylation at these three sites and the shuttling of 2b between nucleus and cytoplasm will be our next subject for further examination.

Phosphorylation at S28 also has a profound effect on the function of 2b. The S28 phosphorylation negatively regulated 2b’s RSS activity and CMV pathogenicity, perhaps due to a decreased level of cytoplasmic 2b (Figs 3–5). These results are in accordance with the previous reports in that 2bs in the cytoplasm play the main role in suppressing RNA silencing [21,22,36]. In addition, the ability of glutamate or aspartate substitution mutants (S28E, S40D/S42D, S40E/S42E and S28E/S40E/S42E) to suppress RNA silencing was reduced or totally impaired, suggesting that phosphorylation at those three serine residues can compromise 2b’s RSS activity (Fig 4B). However, we believe that the S28 amino acid residue is more influential than S40 and S42 in the regulation of 2b’s RSS activity via phosphorylation because it has a great effect on 2b’s subcellular localization. This belief is based on the observation that the 2b’s RSS activity was enhanced by an alanine substitution at S28 compared to that of wild-type 2b (Fig 4B), while the S40A/S42A mutant, which has a serine at S28, had greatly decreased RSS activity (Fig 4B). The two NLS regions and S42 have already been demonstrated to be important for 2b’s siRNA-binding affinity [21,31,49]. Because phosphorylation of amino acids confers a negative charge on the side chains, phosphorylation in proximity to RNA binding sites negatively affects 2b’s sRNA binding. *In silico* analysis based on the 2b-siRNA binding model suggests that the H-bond formation between 2b and siRNA is destroyed by S42 phosphorylation (S10 Fig), and in fact, our *in vitro* siRNA binding assay using wild-type 2b and its phospho-mimetic mutant (S28E and S28E/S40E/S42E) showed a negative correlation between the phosphorylation of 2b and its siRNA-binding activity (S9 and S11 Figs). These results have similarities with Chen et al. (2008) in that R28 and S42 of TAV 2b, which correspond in position to S28 and S42 of CMV 2b, are predicted to form H-bond with uracil 10 and 15, respectively [49].

The phosphorylation of 2b likely regulates its binding to some components in the RNA silencing pathway. For example, phosphorylation of AGO2 strongly reduces the association between AGO2 and sRNAs in *Caenorhabditi elegans* [40]. While AGO2 is also thought to be translocated to the nucleus by IMP [53,54], it is not yet known whether phosphorylation is really necessary for the translocation of AGO2. However, it is worth noting that AGO2 is similar to 2b in that it loses its ability to bind siRNAs upon phosphorylation.

### The subcellular localization of 2b is determined by a balance of importin/exportin-mediated intracellular transport

The translocation of 2b from the nucleus to the cytoplasm is mediated by exportin in that (i) the treatment by exportin inhibitor, LMB, promoted 2b’s accumulation in the nucleus (Figs 1A and S6) and that (ii) the mutations at the leucine residues in the predicted NES motif induced high levels of 2b accumulation in the nucleus and nucleolus (Figs 1, S2 and S6). Only a few cases have been reported on the nuclear export of plant viral proteins. A mutation in the NES motif of BNYVV p25 induced attenuated symptoms in quinoa plants inoculated with the virus containing the mutant p25 [17]. XPO1-dependent nuclear export of the C4 protein of TLCYnV has been found to be critical for developmental abnormalities in virus-infected *N*. *benthamiana* [16]. On the basis of these observations, plant viruses likely utilize not only nuclear import but also nuclear export to enable their own proteins to function in either the nucleus or cytoplasm.

We were then interested in whether the 2bs that had been transported from the nucleus to cytoplasm could still function as an RSS. We thus analyzed the RSS activity of the mutant L77/85/87A. As shown in Fig 6B, L77/85/87A was highly concentrated in the nucleus and did not efficiently suppress RNA silencing. Therefore, we hypothesized that 2b exported from nucleus might be recycled in the cytoplasm to work again as an efficient RSS. It has been reported that 2bs from subgroup II strains predominantly accumulate in the nucleus and are scarcely detectable in the cytoplasm [36] and that 2b of strain CMV-Q (subgroup II) has very weak RSS activity compared to that of 2b of strain CMV-SD (subgroup I) [55]. These observations thus suggest that the presence or absence of the NES site is the major factor underlying the RSS characterizations of CMV subgroups I and II.

The NES-amino acid substitution mutant, L77/85/87A showed very low RSS activity while some C-terminal deletion mutants without NES had RSS activity comparable to that of wild-type 2b. To elucidate this discrepancy, we found a key in the results of our 2b’s RSS activity assay using a series of C-terminal deletion mutants. The RSS activity suddenly recovered to a level similar to that of wild-type 2b just between L77/85/87A(1–87) and L77/85/87A(1–100); we thus considered that 2b’s siRNA-binding activity might be higher in the former than in the latter. We then conducted *in silico* analysis to predict 3D structure models between siRNA and those deletion mutants. As shown S15 Fig, siRNA can make a complex more stably with L77/85/97A(1–87) than with L77/85/97(1–100), based on the H-bond distance and contact surface area between the two molecules. Considering that 2b’s RSS activity in the agroinfiltration assay must be mainly that of cytoplasmic 2b before it is transported to the nucleus, it is conceivable that the C-terminal deletion mutants without NES could exhibit high RSS activity before they are translocated to the nucleus, and that relatively low RSS activity of 2bs with NES including wild-type 2b will be compensated by that of the recycled 2b exported from the nucleus. Although this hypothesis must be further examined, we believe that we could provide one possible explanation for the discrepancy on the 2b’s RSS activity between the C-terminal deletion mutants and L77/85/87A.

In addition to importin and exportin, 2b also interacts with AGOs and other host proteins including plant catalase 3 (CAT3) and rgs-CaM [28,33,56,57]. Duan et al. (2012) previously suggested that the 2b–AGO interaction may affect the intranuclear localization of both proteins [37]. Because 2b’s NLSs overlap with the sRNA binding site, the abundance of sRNAs may affect the phosphorylation of 2b by host kinase(s) and, thus, the 2b–IMP interaction. Therefore, intracellular localization of 2b will be determined by a balance in the accumulation levels among sRNAs, importins, exportins and other host 2b-binding proteins.

### 2b may be recycled to function as an efficient RSS in the nucleus and the cytoplasm by dephosphorylation

When we analyzed the level of phosphorylated 2b in the nuclear-rich fraction, the phosphorylated L77/85/87A was very low compared to the wild-type 2b (Fig 7), suggesting that 2b might be dephosphorylated in the nucleus. In the nucleus, there are various Ser/Thr protein phosphatases that regulate phosphoproteins in the nucleus [58–62]. After 2b is dephosphorylated in the nucleus, it might then be exported from the nucleus to the cytoplasm for recycling. On the other hand, considering that the phospho-mimetic mutant 2b (S28E and S28E/S40E/S42E) lost its sRNA-binding ability (S9 and S11 Figs), the dephosphorylation of 2b might facilitate 2b binding to sRNAs in the nucleus. These observations are also consistent with a previous report that 2b can bind AGO4-related 24-nt siRNAs in the nucleus to interfere with RNA-dependent DNA methylation (RdDM) [33].

On the other hand, we also demonstrated that S28 phosphorylation may be necessary for 2b’s IMP-mediated nucleolar localization (Fig 3). Among the few studies on IMP-mediated nucleolar transport of plant viral proteins, one is the potato mop-top virus triple gene block1 (TGB1) protein, which interacts with two NbIMPα proteins to be translocated into the nucleolus in *N*. *benthamiana* [63], suggesting that IMPα is necessary for the protein transport into the nucleolus. The NLS-mediated nuclear/nucleolar localization of the potato virus A viral genome-linked protein (VPg), which is an RSS, was found to be important for interacting with the protein, suppressor of gene silencing 3 [64,65].

Although the main function of the nucleolus is the synthesis of ribosomal components [66,67], accumulating evidences suggest that the nucleolus is also involved in other cellular functions including gene silencing (reviewed by Kalinina et al. 2018 [68]). In addition to ribosomal components, transfer RNAs, small nucleolar RNAs (snoRNAs) and some mature miRNA precursors are present in the nucleolus [69,70]; some miRNAs are processed from snoRNA precursors [71,72]. snoRNA-derived sRNAs were reported to be associated with AGOs in *Arabidopsis* and human [73,74]. These results thus suggest that 2b may function as an RSS not only in the nucleus but also in the nucleolus by regulating its own phosphorylation.

### Similarities between 2b and AGOs in their subcellular localization and interactions

Bologna et al. (2018) demonstrated that AGO1 of *Arabidopsis* has both NLS and NES motifs, and its transport from the nucleus is mediated by XPO1 [39]. They proposed a revised model of the miRNA pathway in plants; mature miRNAs are loaded on AGO1 in the nucleus, for transport across the nuclear membrane with the help of XPO1 into the cytosol [39]. Thus, AGO1 is shuttled between the nucleus and the cytoplasm to induce miRNA-mediated PTGS. Considering that both 2b and AGO1 depend on XPO1 for their export from the nucleus, they are likely to compete for XPO1. Therefore, 2b may be able to inhibit the step where AGO1 transports mature miRNAs from the nucleus to the cytoplasm by competition for XPOI. This step could be another operational point for 2b to suppress RNA silencing.

On the other hand, a human AGO1 promotes gene expression in human cancer cells by interacting with RNA polymerase II [75]. Such positive gene regulation has also been reported for *Arabidopsis* AGO1 in response to hormones and biotic/abiotic stresses [38]. Therefore, it is conceivable that 2b interacts with AGO1 in the nucleoplasm to negatively regulate transcription of the genes related to some antiviral responses. In addition, 2b also interacts with AGO4 and AGO4-related 24-nt siRNAs, which work in RdDM [30,33]. Pontes et al. (2006) previously reported that the core players of RdDM—RNA-dependent RNA polymerase 2, Dicer-like 3, AGO4, the chromatin remodeler and the *de novo* cytosine methyltransferase—are all colocalized with siRNAs in the nucleolus [76], suggesting that some processes of siRNA generation and RISC formation may occur in the nucleolus. Therefore, there is a good possibility that 2b also works as an RSS in the nucleolus. We believe that 2b is shuttled between the nucleolus and non-nucleolar space in the nucleus, so that 2b can chase AGOs and sRNAs wherever they go in the nucleus.

## Conclusions

**Fig 8:**
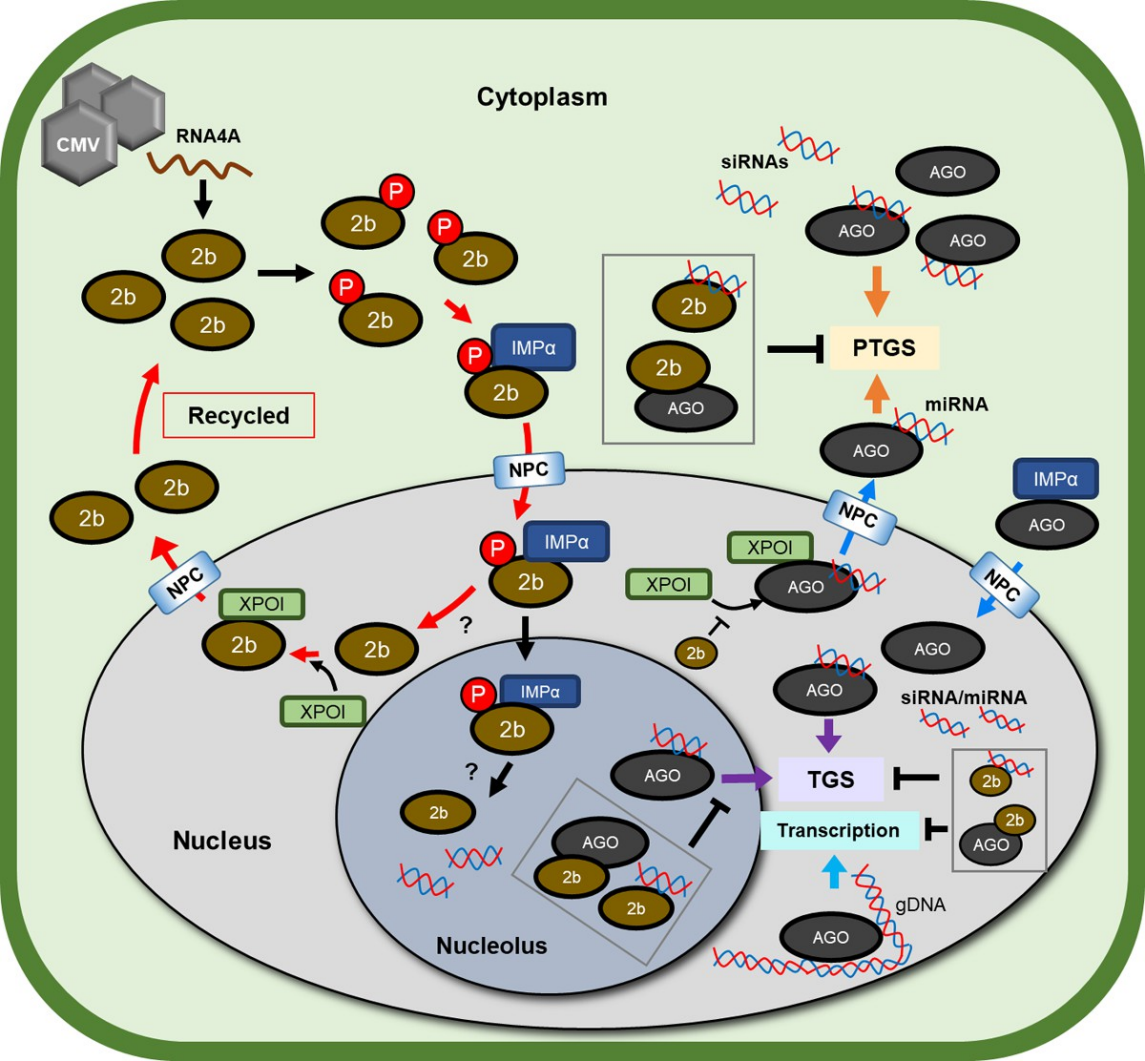
Summary of possible interactions between 2b and players in the RNA silencing pathway in cells. Subcellular trafficking of 2b and its operation points to suppress RNA silencing are shown as red bold arrows. Cytoplasmic 2b is first phosphorylated by a host kinase. The phosphorylated 2b can interact with IMPα, and the 2b/IMPα complex passes through the nuclear pore complex (NPC) into the nucleus and possibly into the nucleolus. AGOs are also imported into the nucleus and nucleolus in a similar IMP-mediated manner. 2b may be recycled after dephosphorylation by host phosphatase(s) in the nucleus or nucleolus; dephosphorylated 2bs will be exported to the cytoplasm in a XPOI-dependent manner, while they can interact with AGOs and sRNAs either in nucleus or nucleolus [37]. AGOs are also exported to the cytoplasm in a XPOI-dependent manner, transporting mature miRNAs to the cytoplasm [39]. Therefore, 2bs seem to shuttle between the nucleus and the cytoplasm as they efficiently track targets such as AGOs and siRNAs.

On the basis of our findings and those previously reported, we summarize 2b’s behavior in nucleocytoplasmic shuttling controlled by phosphorylation/dephosphorylation and two signal motifs (NLS and NES) on 2b and how 2b interacts with other components in the RNA silencing pathway (Fig 8). In this study, we demonstrated that the dynamic nucleocytoplasmic shuttling of 2b is important for its RSS activity and that 2b can efficiently find its targets, AGOs and sRNAs in the nucleus and the cytoplasm. For 2b to work as an efficient RSS, it uses both phosphorylation and dephosphorylation to move back and forth between the nucleus/nucleolus and the cytoplasm, creating a sustainable recycling system (Fig 8, red-bold arrows). Here, we are intrigued by the similarities between 2b and AGOs in their intracellular trafficking: (i) 2b and AGO1 are both imported into the nucleus and nucleolus, (ii) both proteins have sRNA-binding ability, which is negatively regulated by phosphorylation, and (iii) both are exported from the nucleus to the cytoplasm in an XPOI-dependent manner. In this respect, we presume that 2b and AGOs may have co-evolved through their interactions in the RNA silencing pathway.

## Materials and methods

### Plant materials and virus inocula

*N*. *benthamiana* and *N*. *tabacum* plants were grown at 25°C with 16 h light, 8 h dark. *N*. *benthamiana* plants were used for agroinfiltration assays and symptom observations. *N*. *tabacum* plants were inoculated with the CMV constructs containing the mutant 2bs for *in vivo* phosphorylation assays. Transcripts of CMV RNAs 1 and 3, and RNA 2 containing mutant 2bs were synthesized *in vitro* as described before [77]. Plants were mechanically inoculated with a mixture of three transcripts (RNAs 1–3) using carborundum [22].

### Construction of 2b recombinants and CMV infectious vectors

The mutant 2b constructs, which have point mutations at possible phosphorylation sites, were created by recombinant PCR using the primers listed in S1 Table, and then inserted between the StuI and SpeI sites in the CMV-H1 vector to generate RNA 2 harboring the mutant 2b [47]. All the mutant 2b clones in CMV-H1 were sequenced to confirm their point mutations before use. Additional sequencing analyses were conducted to check whether the introduced 2b mutations were still maintained (S16 Fig).

### Prediction of NES in 2b proteins

The NES motif in the 2b proteins of four CMV strains were analyzed by NetNES 1.1 Server (http://www.cbs.dtu.dk/services/NetNES/). Residues with a NES score higher than the threshold and its nearby amino acids, where a peak score in the HMM or artificial neural network (NN) was found, were predicted as the NES motif.

### *Agrobacterium*-mediated transient overexpression

For the transient expression of 2b, the 2b genes were integrated into pBE2113 between the XbaI and SacI sites. For subcellular localization studies, the 2b-GFP and histone 2B-DsRed (H2B-DsRed) constructs were generated using the primers listed in S1 Table. *A*. *tumefaciens* strain KYRT1 was transformed with the vector construct by the conventional freeze–thaw method. The transformed KYRT1 was cultured in yeast extract peptone (YEP) medium (1% yeast extract, 1% peptone and 0.5% NaCl, w/v) containing 10 μM of acetosyringone. The harvested cells were resuspended in the *Agrobacterium* resuspension buffer (10 mM MgCl_2_, 10 mM MES and 0.2 mM acetosyringone) at ~1.0 optical density at 600 nm (OD_600_), then allowed to sit for 2–3 h, *N*. *benthamiana* leaves were then infiltrated with the inoculum (100–200 μL for each patch) using a 1-mL syringe without a needle. H2B-DsRed was co-expressed by agroinfiltration as a nuclear marker. Leaves were examined for GFP signal at 2 days post agroinfiltration by fluorescence microscopy (Leica DMI 6000B with filter block L5 containing a 480/40 nm excitation filter, a 505 nm dichroic mirror and a 527/30 nm barrier filter for GFP fluorescence), and images were taken using Leica LAS AF software.

### Leptomycin B treatment

The leptomycin B (LMB) treatment was carried out following the method described in Mei et al. (2018) [16] with slight modifications. GFP-tagged 2bs were overexpressed by agroinfiltration in *N*. *benthamiana*. The leaf tissues in the agroinfiltrated patches were infiltrated with LMB (40 nM) at 2 days post agroinfiltration, and the leaves were excised at 4 h after the LMB treatment for microscopic observation. The subcellular localization of 2b-GFP was then observed by fluorescence microscopy (Leica DMI 6000B).

### Detection of 2b in the nuclear-rich fraction

GFP- or FLAG-tagged 2b was expressed in *N*. *benthamiana* leaves after agroinfiltration, and the nuclear-rich fractions were isolated as previously described [35]. 2b-GFP or 2b-FLAG in the nuclear-rich fraction was then detected using western blot analysis with anti-GFP antibodies or anti-FLAG antibody. Histone H3 (H3) was detected using anti-H3 antibodies as a loading control. To compare the levels of phosphorylated 2b in the nuclear-rich fraction, the phosphoproteins in the nuclear-rich fraction were concentrated using the TALON PMAC phosphoprotein enrichment kit (Clontech, Tokyo, Japan) and the manufacturer’s instruction (S5 Fig); the phosphorylated 2b was detected by western blot analysis using anti-2b antibodies.

### *In vitro* phosphorylation assay

Seven putative phosphorylation sites including one threonine and six serines were predicted by the NetPhos v2.0 program (http://www.cbs.dtu.dk/services/NetPhos-2.0/). Truncated mutant 2bs were generated by PCR and inserted between the EcoRI and SalI restriction enzyme sites in pMAL-c2x (NEB, Beverly, MA, USA). The predicted phosphorylation sites were replaced with alanine using the primer pairs listed in S1 Table. Full-length mutant 2bs were also created by recombinant PCR using the primer pairs listed in S1 Table, then cloned into pMAL-c2x using the EcoRI and SalI restriction enzyme sites. BL21 *E*. *coli* competent cells were transformed with the pMAL-c2x vectors containing the mutant 2bs. The MBP-2b fusion proteins were expressed in *E*. *coli* and harvested as previously described [47]. The extracted proteins were then purified using the pMAL Protein Fusion and Purification System (NEB, Beverly, MA, USA) according to the instructions. The components in the elution buffer were exchanged with the kinase reaction buffer (10 mM MgCl_2_, 1 mM DTT and 20 mM HEPES [pH 7.5]) using a Slide-A-Lyzer MINI Dialysis Unit (Thermoscientific, Rockford, IL, USA). Purified proteins were incubated in the kinase reaction buffer (total 15 μL reaction volume) containing 37 nM ATP, 0.25% Complete Mini Protease Inhibitor Cocktail (Roche, Basel, Switzerland) and 0.5 μL of protein kinase C (Promega, Madison, WI, USA). The *in vitro* phosphorylated proteins were then separated by SDS-PAGE and detected using the ProQ-Diamond Phosphoprotein Gel Stain kit (Invitrogen, Carlsbad, CA, USA).

### *In vivo* phosphorylation assay

Total protein extracts were prepared from infected leaves of *N*. *tabacum* plants at 6 days post inoculation. Phosphorylated proteins were concentrated using the TALON PMAC phosphoprotein enrichment kit (Clontech) according to the manufacturer’s instructions (S5 Fig). Equal amounts of total protein extracts were loaded on a phosphoprotein enrichment column, then the phosphoproteins were eluted from the column with the included elution buffer. Phosphorylated 2bs were then separated by 14% SDS-PAGE and detected with western blot analysis using anti-2b antibodies [47].

### Co-immunoprecipitation assay

The CDS region of the NbIMPα1 gene (GenBank: ABM05487.1) was amplified, and the FLAG tag (NbIMPα1-FLAG) was added to the C-terminus by RT-PCR using the primer pairs listed in S1 Table. The NbIMPα1-FLAG sequence was then cloned in the pBE2113 binary vector between the XbaI and SacI restriction enzyme sites. The *Agrobacterium* strains KYRT1 carrying the NbIMPα1-FLAG and 2b-GFP constructs were used to co-infiltrate into *N*. *benthamiana* leaves at a 1:1 ratio with OD_600_ = 1.0. Infiltrated leaf patches (0.2 g) were homogenized in 1 mL of the lysis buffer (20 mM HEPES pH 7.5, 1 mM EDTA, 40 mM KCl, 0.1% (v/v) Triton-X 100, 1 mM PMSF, 10% (v/v) glycerol) containing 1X protease inhibitor cocktail (Sigma Aldrich) [78], and the soluble fraction of the homogenates was extracted by two times centrifugation at 10,000 × *g* for 10 min. The total protein extract in the soluble fraction was co-incubated with by shaking. After washing, the bound proteins were eluted (Dynabeads Protein G Immunoprecipitation Kit; Invitrogen). The precipitant was then detected by western blot analysis using anti-GFP antibodies (MBL).

### Detection of 2b in the cytoplasmic fraction

The cytoplasmic fraction was isolated as shown in S14 Fig. Agroinfiltrated *N*. *benthamiana* leaf tissues were homogenized in 5 mL of the extraction buffer (0.35 M sucrose, 50 mM Tris-Cl, pH 7.5, 5 mM MgCl_2_, 2 mM 2-mercaptoethanol) per gram of tissue. Nuclei, plastids and cell wall components were removed by centrifugation at 2500 × *g* for 10 min, 4°C, and the supernatant was then centrifuged at 6000 × *g* for 10 min, 4°C to eliminate mitochondria. The final supernatant after centrifugation was used for immunoprecipitation. Cytoplasmic 2b was detected using the Dynabeads Protein G Immunoprecipitation kit (Invitrogen) with anti-2b antibodies and the manufacturer’s instructions. The precipitant was then detected by western blot analysis using anti-FLAG antibody (Fujifilm).

### Analysis of 2b’s RSS activity

The conventional agroinfiltration assay for RSS activity was conducted as previously described [22]. The GFP and GFP inverted-repeat (GFP-IR) constructs were inserted in the pBE2113 vector between the XbaI and SacI sites. The pBE2113 expression vectors containing 2b, GFP and GFP-IR were introduced into *A*. *tumefaciens* strain KYRT1. Inocula consisted of a combination of *Agrobacterium* cultures carrying a 5:1:5 ratio of GFP, GFP-IR and 2b constructs. *N*. *benthamiana* leaves were agroinfiltrated with bacterial suspensions (~1.0 OD_600_). The suspension of *Agrobacterium* cells that carried either 2b or S28A was serially diluted to OD_600_ = 0.5, 0.25 and 0.13. GFP signals were observed at 2 days post infiltration under UV light. For infiltrating onion epidermis cells, the bacterial resuspensions were adjusted to OD_600_ = 0.2 for each GFP, GFP-IR and 2b construct. GFP fluorescence was observed at 3 days post infiltration in Leica DMI 6000B, and the intensity was calculated using the Leica LAS AF software.

### Quantitative real-time RT-PCR

Total RNAs were isolated using Trizol (Roche) and treated by DNase I to remove DNA contaminants. cDNAs were synthesized using the PrimeScript RT Reagent Kit (Takara, Tokyo, Japan), then used for real-time RT-PCR with the StepOnePlus Real-Time PCR system (Applied Biosystems, Foster City, CA, USA). Primer pair CMV-DET-5/CMV-DET-3 was used to quantify the levels of CMV RNAs 3 and 4; primer pair Nb-L23-5/Nb-L23-3 was used as a reference for amplification of a partial mRNA sequence of 60S ribosomal protein L23 in *N*. *benthamiana* (S1 Table). For amplification of GFP transcripts, primer pair S65T-5-168/S65T-3-168 was used (S1 Table).

## Supporting information

S1 Fig Prediction of the NES motif in the 2b proteins. (A) Nuclear localization signals (NLSs) and a predicted nuclear export signal (NES) of 2b proteins. The amino acid positions are indicated above the alignment. (B) NES in proteins Y2b, Y/HL2b, HL2b and R2b was predicted using the NetNES 1.1 server. The NES motif was identified when the calculated NES score exceeded the threshold and its adjacent residues, where a peak was found by the HMM score or NN score, were also predicted to be a putative NES [79].
(TIF)

S2 Fig Subcellular localization of Y2b and Y2bΔC that lacks the C-terminal 29 amino acids including NES in *N*. *benthamiana*. (A) CMV RNA2 constructs containing Y2b-DsRed and Y2bΔC-DsRed. The *DsRed* gene was amplified and fused to the 3′ end of the 2b gene by PCR. (B) Comparison of the subcellular localization between Y2b-DsRed and Y2bΔC-DsRed. *N*. *benthamiana* was coinoculated with the recombinant RNA 2 and RNA 1 and 3 of CMV-Y to express Y2b-DsRed and Y2bΔC-DsRed. DsRed florescence was observed and images captured with an epifluorescence microscope (Leica DMI 6000B) at 7 dpi. Scale bar: 50 μm.
(TIF)

S3 Fig *In vitro* phosphorylation of 2b protein of CMV. (A) Putative phosphorylation sites and NLSs on the 2b protein. Amino acid positions are indicated above the alignments. Seven residues (red boxes) were predicted as phosphorylation sites by the NetPhos v2.0 program. The T9, S40 and S42 residues were previously predicted as putative CKII phosphorylation sites [29]. (B) *In vitro* phosphorylation of the MBP-2b fusion proteins (MBP-HL2b and MBP-Y/HL2b). Purified MBP (negative control), MBP-HL2b and MBP-Y/HL2b were treated with PKC. Phosphorylated proteins were detected by phosphoprotein gel staining (upper panel); the loading control was stained with Coomassie brilliant blue (CBB).
(TIF)

S4 Fig Inoculation of CMV-H1 vectors for *in vivo* phosphorylation assay. (A) Schematic map of the CMV-H1 RNA 2 vector. CMV-H1 RNA 2 in which the entire 2b ORF was deleted and a MCS was inserted [48]. (B) CMV symptoms on tobacco plants at 7 dpi induced by CMVs containing Y/HL2b, Y/HL[A], [A]S28 and [A]S40/S42. (C) Proteins were extracted from the leaf tissues of infected plants and subjected to western blot analysis using anti-CP and anti-2b antibodies. RuBisCo large subunit is shown as a loading control.
(TIF)

S5 Fig Isolation of *in vivo* phosphorylated 2b using the phosphoprotein enrichment kit. The initial soluble protein fractions extracted from CMV-infected *N*. *tabacum* leaves were loaded on a phosphoprotein affinity column. The PMAC resin is highly selective for the phosphates on the proteins, allowing other proteins and contaminants to pass through the column and go into the flow-through. The phosphorylated proteins are eventually eluted from the column with the elution buffer supplied by the manufacturer, and 2b was detected by western blot analysis using anti-HL2b antibodies.
(TIF)

S6 Fig Effect of a mutation in NES on the subcellular localization of S28E. The GFP-tagged 2b mutants were co-expressed with H2B-DsRed in *N*. *benthamiana* by agroinfiltration. LMB (40 nM) was infiltrated into the leaves at 2 days post agroinfiltration. GFP florescence was observed at 4 h after LMB treatment using Leica DMI 6000B. Scale bar: 50 μm (white), 5 μm (yellow).
(TIF)

S7 Fig PVX virus-induced gene silencing (VIGS) of NbIMPα1 and NbIMPα2 genes. (A) Alignment of AtIMPα1 and two IMP orthologues (NbIMPα1 and NbIMPα2) of *N*. *benthamiana*. The amino acid positions are indicated above the alignment. (B and C) Real-time RT-PCR quantification of NbIMPα1 and NbIMPα2 transcripts in the silenced *N*. *benthamiana* plants. A partial sequence of either NbIMPα1 or NbIMPα2 was cloned into the PVX vector in a reverse orientation (PVX-IMPα1 or PVX-IMPα2). *N*. *benthamiana* plants were co-inoculated with PVX-IMPα1 and PVX-IMPα2 (PVX-IMPα1+2) to simultaneously silence NbIMPα1 and NbIMPα2. Total RNA extracts from the infected leaves were analyzed at 15 dpi. Mean values (±SE) (*n* = 4) are fold-changes calculated when the value of PVX is set to 1.0, and the values were analyzed on log-transformed data for a significant difference using Student’s *t*-test (**P < 0.01, ****P < 0.0001). (D) The PVX RNA levels were quantified by real-time RT-PCR. Mean values (±SE) (*n* = 5) are fold-changes calculated when the value of PVX is set to 1.0, and the values were analyzed on log-transformed data for a significant difference using Student’s *t*-test (*P* > 0.05).
(TIF)

S8 Fig Effect of NbIMPα silencing on nuclear localization of 2b in *N*. *benthamiana*. (A–C) 2b-GFP was overexpressed by agroinfiltration in PVX- or PVX-IMP-infected *N*. *benthamiana* at 15 dpi. GFP fluorescence was observed at 2 days post-infiltration using a Leica DMI 6000B (A). Scale bar: 50 μm. (B) Western blot of 2b-GFP in the nuclear-rich fraction detected using anti-GFP antibodies. The numbers above the blot are five plants replicates. H3 was detected using anti-H3 antibodies as a loading control. (C) The relative 2b-GFP/H3 ratio was densitometrically calculated using Multi Gauge Software (Fujifilm) and indicated as a fold-change value. Mean values (±SE) were compared on log-transformed data for significant differences using Student’s *t*-test (***P < 0.001). (D–F) The free GFP was expressed as a control by agroinfiltration in PVX- or PVX-IMP-infected *N*. *benthamiana*. (D) Subcellular localization of the GFP control at 2 days post-infiltration. (E) Western blot analysis for GFP and H3 using the nuclear-rich fractions. The numbers above the blot are four plants replicates. (F) Relative GFP/H3 ratio was densitometrically calculated using Multi Gauge Software (Fujifilm) and indicated as a fold-change. Mean values (±SE) were compared on log-transformed data for a significant difference using Student’s *t*-test. (G–I) Effect of IMPα silencing on the subcellular localization of the 2b-R46C mutant (R46C), which has very weak RSS activity. The R46C-fused GFP construct (R46C-GFP) was overexpressed by agroinfiltration in the PVX- or PVX-IMP-infected *N*. *benthamiana*. (G) Subcellular localization of R46C at 2 days post-infiltration. (H) Western blot of R46C-GFP and H3 detected as described above. The numbers above the blot are five plants replicates. (I) Relative 2b-GFP/H3 ratio was densitometrically calculated using the Multi Gauge Software (Fujifilm) and indicated as fold-changes. Mean values (±SE) were compared on log-transformed data for a significant difference using Student’s *t*-test (***P < 0.01).
(TIF)

S9 Fig sRNA-binding ability of 2b and phospho-mimetic 2b. MBP was fused to the N-terminal region (46 amino acid region) of 2b (MBP-2b). The recombinant proteins were synthesized in *E*. *coli* and column-purified. The purified proteins were then co-incubated with biotin-labeled 21-nt siRNA (5′-UUGCUCAACAGUAUGGGCAUU-biotin-3′). The recombinant proteins were co-precipitated by the biotin–siRNA interaction using Dynabeads M-280 Streptavidin (Invitrogen) and detected in 10% polyacrylamide gel by CBB staining. MBP was used as a control.
(TIF)

S10 Fig Effect of phosphorylation sites of 2b on the 2b-siRNA binding in the ICM software-predicted model structures. The crystallography of the TAV 2b protein (PDB ID: 2ZI0) of TAV has been reported [49]; data are available in the Research Collaboratory for Structural Bioinformatics Protein Data Bank (RCSB PDB) [80]. The protein structure of TAV 2b was used for homology modeling of CMV 2b. Structures of wild-type CMV 2b and its mutants were modeled by I-TASSER v5.1 (https://zhanglab.ccmb.med.umich.edu/I-TASSER/) [81]. The 2b structures in the state of siRNA binding are representative low-energy conformations after the global torsion was minimized using ICM-Pro. The predicted structures were visualized and matched with TAV 2b using ICM-Pro (Molsoft, San Diego, CA, USA), and those structures are low-energy conformations after the global torsion were minimized in ICM-Pro. H-bond (H) formation (red-dashed circles) was examined using ICM-Pro and UCSF chimera [82] (A). The distances between the alpha/beta carbon (C_α_/C_β_) of S42 and the phosphate oxygen of cytosine 14 (C14PO) of siRNA were calculated using UCSF chimera (B). 2bs with some RSS activity (Fig 4) are underlined. The H-bond in S40A/S42A was not created because S42 was replaced with an alanine residue and thus does not have a hydroxyl group for H-bond formation. Note that 2b’s RSS activity seems to be correlated to the distance between the hydroxyl group of the serine residue 42 of 2b and the phosphate oxygen of the cytosine residue 14 of siRNA that are involved in the H-bond. The importance of this H-bond was also suggested by Nemes et al. (2017) [31].
(TIF)

S11 Fig sRNA binding of 2b, S28E and S77/85/87A. MBP was fused to the N-terminal of 2b, S28E and S77/85/87A. The recombinant proteins were synthesized in *E*. *coli* and column-purified. The co-precipitation of biotin-labeled siRNA and the *E*. *coli*-synthesized proteins were treated as described in S9 Fig. The amounts of the recombinant proteins were compared between 2b and S28E (A) or between 2b and S77/85/87A (B). The MBP-fused proteins were detected in a 10% polyacrylamide gel by CBB staining. The black arrowheads indicate 2b fused with a truncated form of MBP, which is often generated in *E*. *coli* [47].
(TIF)

S12 Fig Disease severity in plants inoculated with CMVs expressing the mutant 2bs. (A) Mutants with alanine substitutions at various putative phosphorylation sites were created, and CMV RNA 2 constructs with those 2b mutants were prepared by inserting the 2b fragments into the CMV H1 vector. (B) Symptom severity on *N*. *benthamiana* plants inoculated with the CMV constructs carrying the various 2b mutants. [A]S28, [A]S40/S42 and [A]S28/S40/S42 were created using the primers in S1 Table. The mutant 2b backbone with alanine residues at all the putative phosphorylation sites is shown as “[A]”. Symptoms were observed at 11 dpi. (C, D) Relative accumulation level of CMV RNA 3 in systemically infected upper leaves determined by real-time RT-PCR using primer pairs listed in S1 Table. The values of fold-changes were analyzed on log-transformed data by Student’s *t*-test (***P < 0.001, ****P < 0.0001). (E) Symptoms on upper leaves of the plants 21 days after inoculation (dpi) with either CMV:[A]S40/S42 or CMV:[A]S28/S40/S42. (F) Relative accumulation levels of CMV RNA 3 and 4 in the plants infected with CMV:[A]S40/S42 or CMV:[A]S28/S40/S42 at 21 dpi. Tissues were collected from the leaves in S9E Fig for quantitative RT-PCR. Means were compared on log-transformed data for a significant difference using Student’s *t*-test (**P < 0.01).
(TIF)

S13 Fig Effect of C-terminal deletion on 2b’s RSS activity. (A) RSS activity of 2b, S77/85/87A and the C-terminal deletion mutants. A series of C-terminal deletion mutants were created using S77/85/87A as the background (B), and their RSS activity was examined as described in Fig 6B. (C) The expression levels of GFP and 2b were estimated by western blot analysis using anti-GFP and anti-2b antibodies, respectively. Red arrows indicate 2b and the 2b mutants. RuBisCo large subunit is shown as a loading control.
(TIF)

S14 Fig Experimental system to detect cytoplasmic 2b in *N*. *benthamiana*. Agroinfiltrated leaf tissues were homogenized in extraction buffer (0.35 M sucrose, 50 mM Tris-Cl, pH 7.5, 5 mM MgCl_2_, 2 mM 2-mercaptoethanol), and the cytoplasmic fraction was separated by a series of differential centrifugations. The supernatant after the final centrifugation was used for western blot analysis.
(TIF)

S15 Fig *In silico* analysis to compare the interface residues in the siRNA-2b complexes. (A) Based on the 2b-siRNA binding model in S10 Fig, the 3D structures of the siRNA-2b complexes using wild-type 2b and the C-terminal deletion mutants were constructed, and the global energy of each complex was optimized by minimizing torsion in ICM-Pro. The H-bond (H) between S42 and cytosine 14 (C14) (black-dotted circles) were visualized using both UCSF Chimera and ICM-Pro programs. The molecular surface areas of 2b and siRNA were generated in ICM-Pro and their surface contact area were analyzed. The regions in the red-dotted circles and black arrows (indicated as 1–3) are the points where the overlapped areas were clearly different between 2b(1–87) and 2b(1–100). (B) The distance of the H-bond between the hydroxyl group of S42 and the phosphate of C14 was measured in the UCSF Chimera. (C) The interface areas shared by 2b and siRNA were calculated using ICM-Pro.
(TIF)

S16 Fig Sequencing analysis to validate the 2b’s mutations in infected plants. To confirm that the 2b’s mutations are maintained during viral infection, the entire 2b sequences were amplified by RT-PCR from the infected plants at 15 dpi. Their sequences were analyzed by the BigDye terminator method. The wave raw data of the sequencing analyses were shown. Each codon of the mutations is indicated in red bars.
(TIF)

S1 Table Primers used for cloning and real-time PCR. (PDF)
