# Supplementary figures and images for: Importin/exportin-mediated nucleocytoplasmic shuttling of cucumber mosaic virus 2b protein is required for 2b’s efficient suppression of RNA silencing
Source: PLoS Pathog. 2022 Jan 26;18(1):e1010267. doi: 10.1371/journal.ppat.1010267 (PMC8820599; doi:10.1371/journal.ppat.1010267)

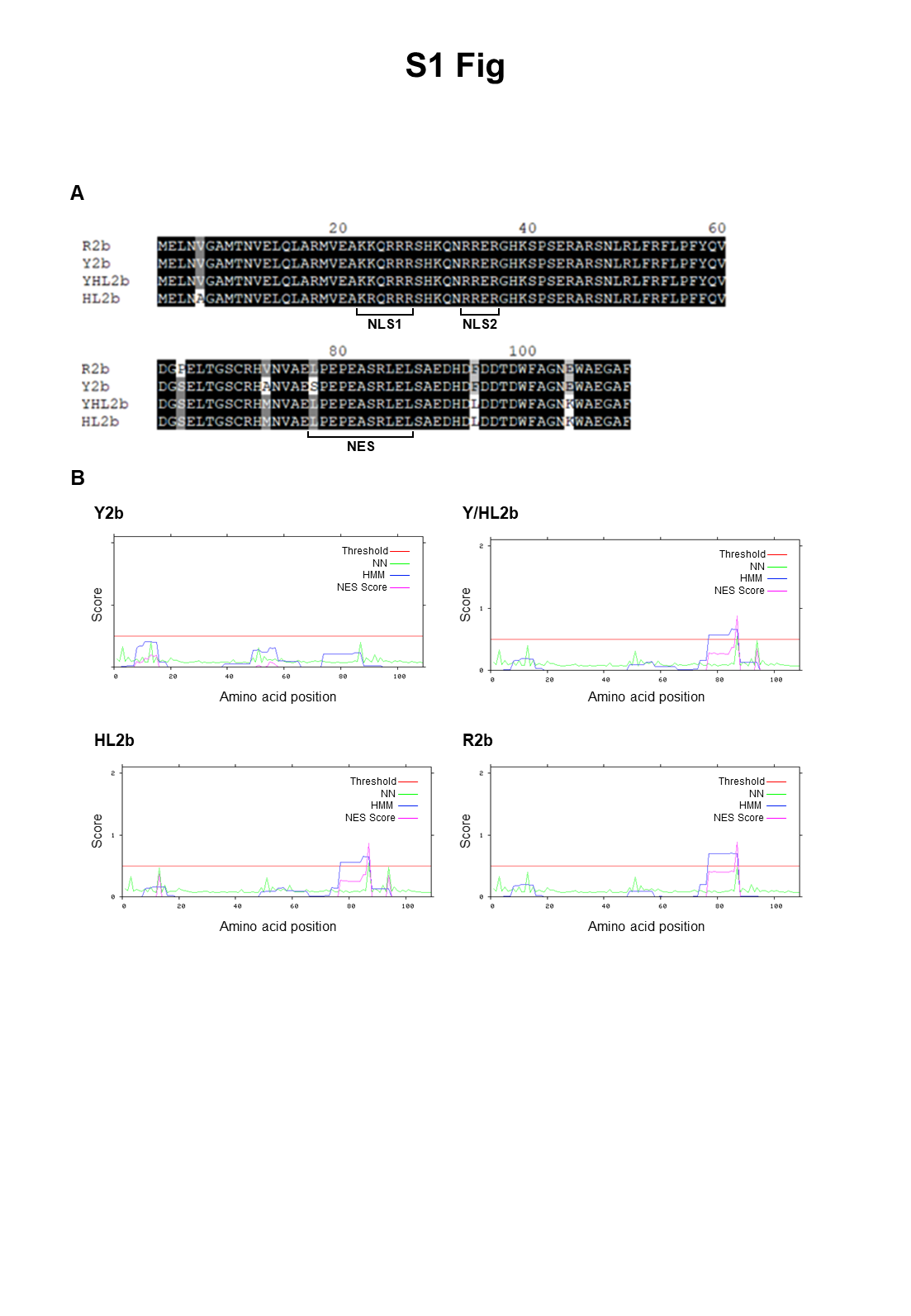

Supplement: S1 Fig — (A) Nuclear localization signals (NLSs) and a predicted nuclear export signal (NES) of 2b proteins. The amino acid positions are indicated above the alignment. (B) NES in proteins Y2b, Y/HL2b, HL2b and R2b was predicted using the NetNES 1.1 server. The NES motif was identified when the calculated NES score exceeded the threshold and its adjacent residues, where a peak was found by the HMM score or NN score, were also predicted to be a putative NES [79]. (TIF) [file ppat.1010267.s001.tif]

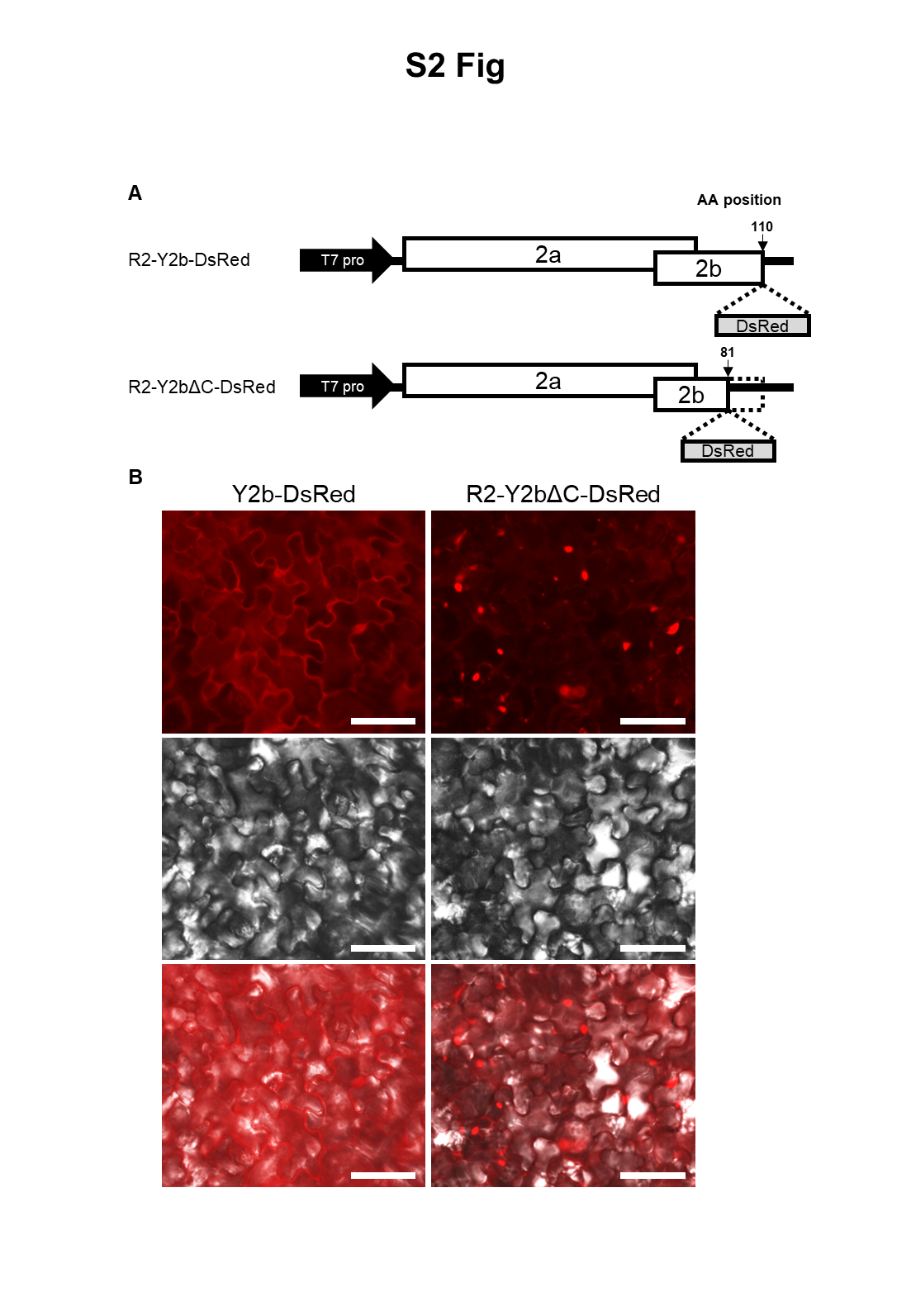

Supplement: S2 Fig — (A) CMV RNA2 constructs containing Y2b-DsRed and Y2bΔC-DsRed. The DsRed gene was amplified and fused to the 3′ end of the 2b gene by PCR. (B) Comparison of the subcellular localization between Y2b-DsRed and Y2bΔC-DsRed. N. benthamiana was coinoculated with the recombinant RNA 2 and RNA 1 and 3 of CMV-Y to express Y2b-DsRed and Y2bΔC-DsRed. DsRed florescence was observed and images captured with an epifluorescence microscope (Leica DMI 6000B) at 7 dpi. Scale bar: 50 μm. (TIF) [file ppat.1010267.s002.tif]

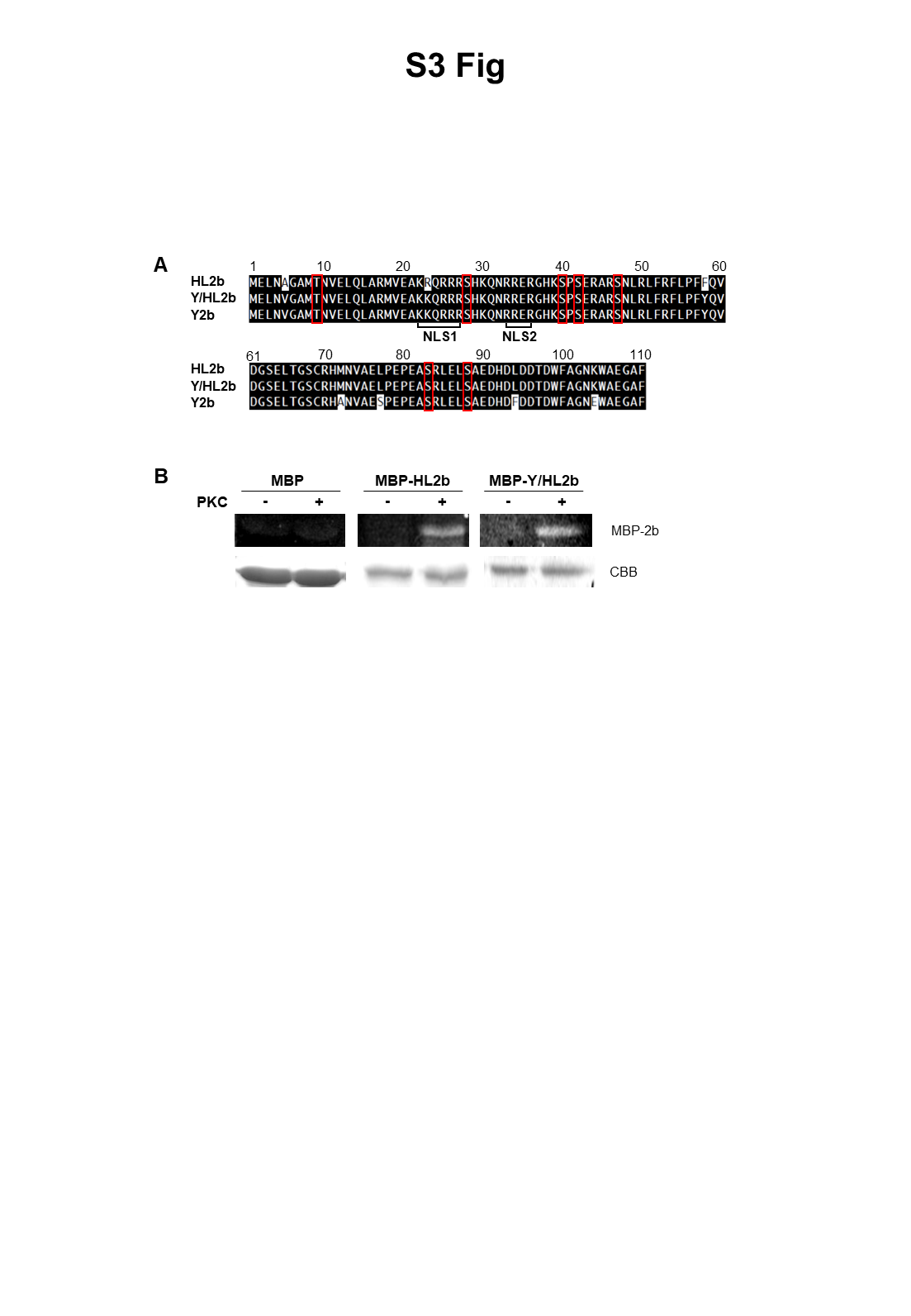

Supplement: S3 Fig — (A) Putative phosphorylation sites and NLSs on the 2b protein. Amino acid positions are indicated above the alignments. Seven residues (red boxes) were predicted as phosphorylation sites by the NetPhos v2.0 program. The T9, S40 and S42 residues were previously predicted as putative CKII phosphorylation sites [29]. (B) In vitro phosphorylation of the MBP-2b fusion proteins (MBP-HL2b and MBP-Y/HL2b). Purified MBP (negative control), MBP-HL2b and MBP-Y/HL2b were treated with PKC. Phosphorylated proteins were detected by phosphoprotein gel staining (upper panel); the loading control was stained with Coomassie brilliant blue (CBB). (TIF) [file ppat.1010267.s003.tif]

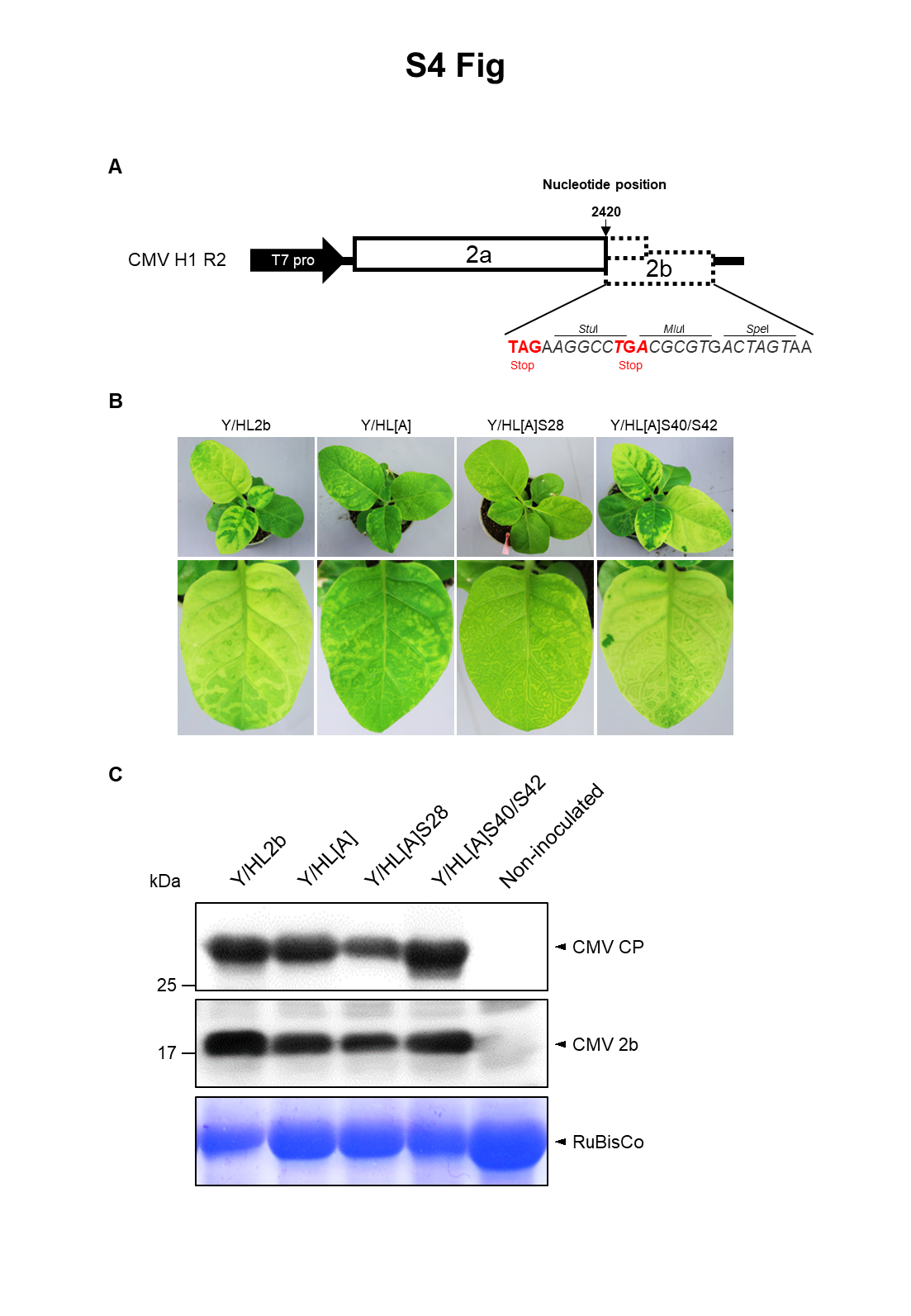

Supplement: S4 Fig — (A) Schematic map of the CMV-H1 RNA 2 vector. CMV-H1 RNA 2 in which the entire 2b ORF was deleted and a MCS was inserted [48]. (B) CMV symptoms on tobacco plants at 7 dpi induced by CMVs containing Y/HL2b, Y/HL[A], [A]S28 and [A]S40/S42. (C) Proteins were extracted from the leaf tissues of infected plants and subjected to western blot analysis using anti-CP and anti-2b antibodies. RuBisCo large subunit is shown as a loading control. (TIF) [file ppat.1010267.s004.tif]

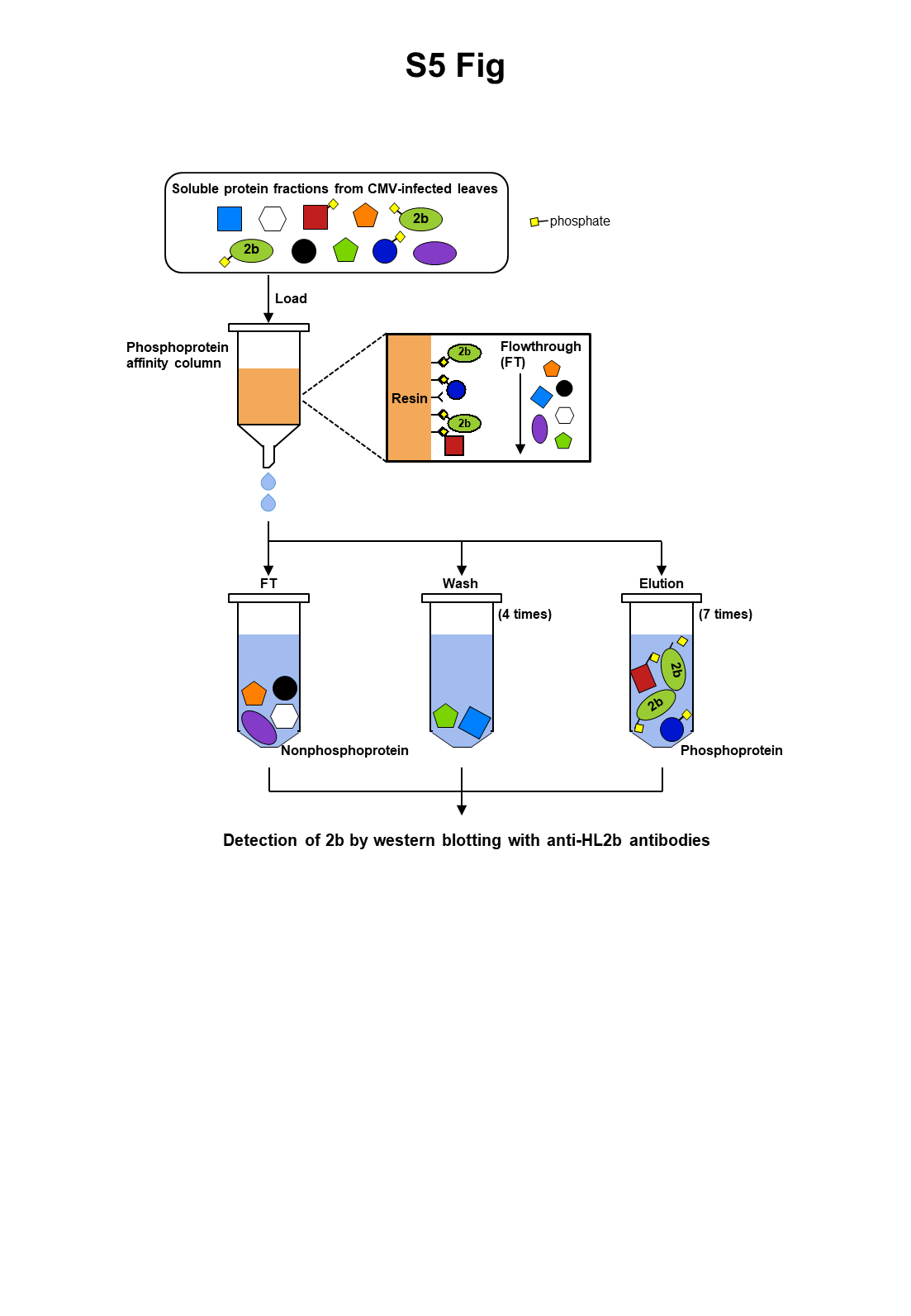

Supplement: S5 Fig — The initial soluble protein fractions extracted from CMV-infected N. tabacum leaves were loaded on a phosphoprotein affinity column. The PMAC resin is highly selective for the phosphates on the proteins, allowing other proteins and contaminants to pass through the column and go into the flow-through. The phosphorylated proteins are eventually eluted from the column with the elution buffer supplied by the manufacturer, and 2b was detected by western blot analysis using anti-HL2b antibodies. (TIF) [file ppat.1010267.s005.tif]

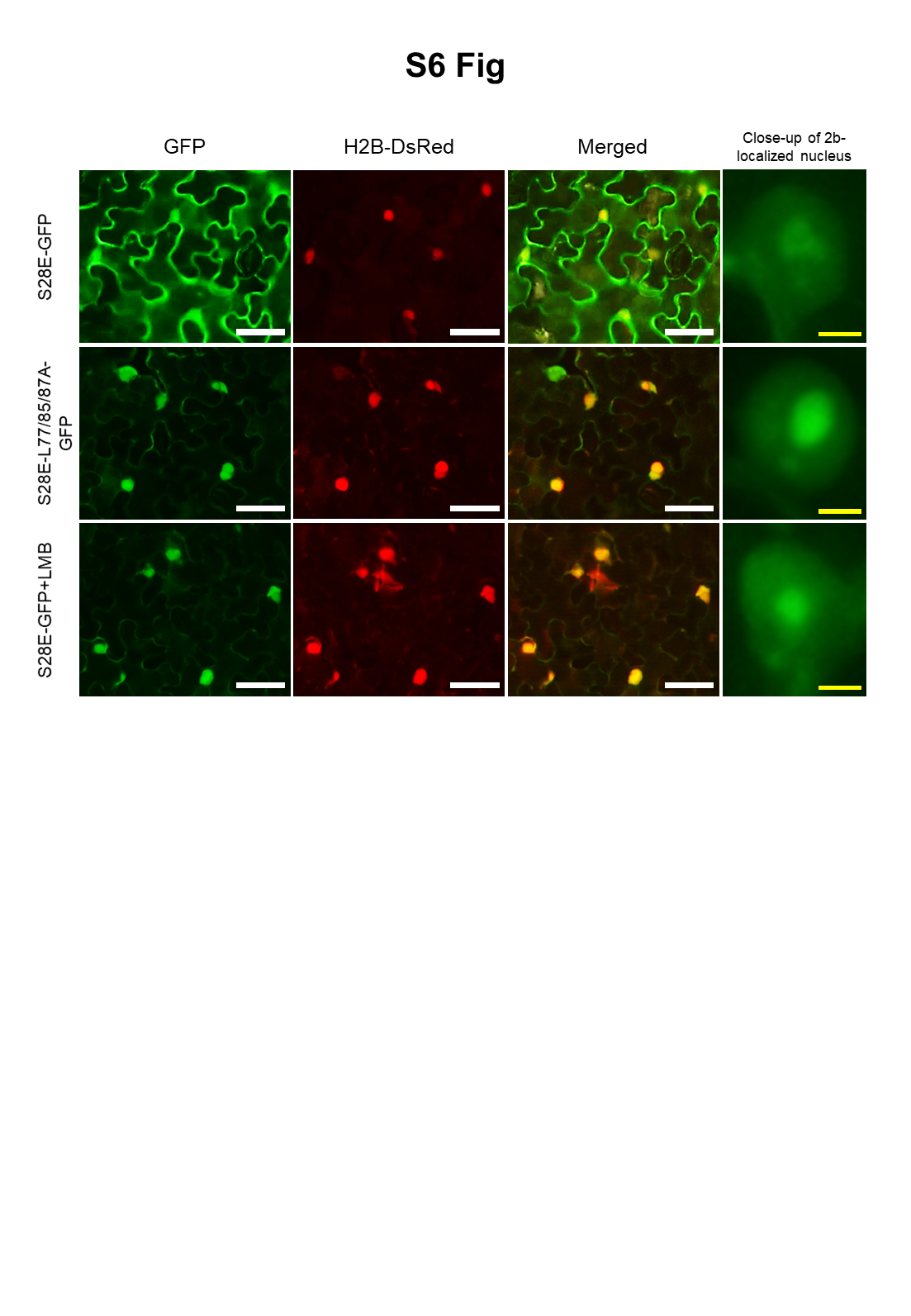

Supplement: S6 Fig — The GFP-tagged 2b mutants were co-expressed with H2B-DsRed in N. benthamiana by agroinfiltration. LMB (40 nM) was infiltrated into the leaves at 2 days post agroinfiltration. GFP florescence was observed at 4 h after LMB treatment using Leica DMI 6000B. Scale bar: 50 μm (white), 5 μm (yellow). (TIF) [file ppat.1010267.s006.tif]

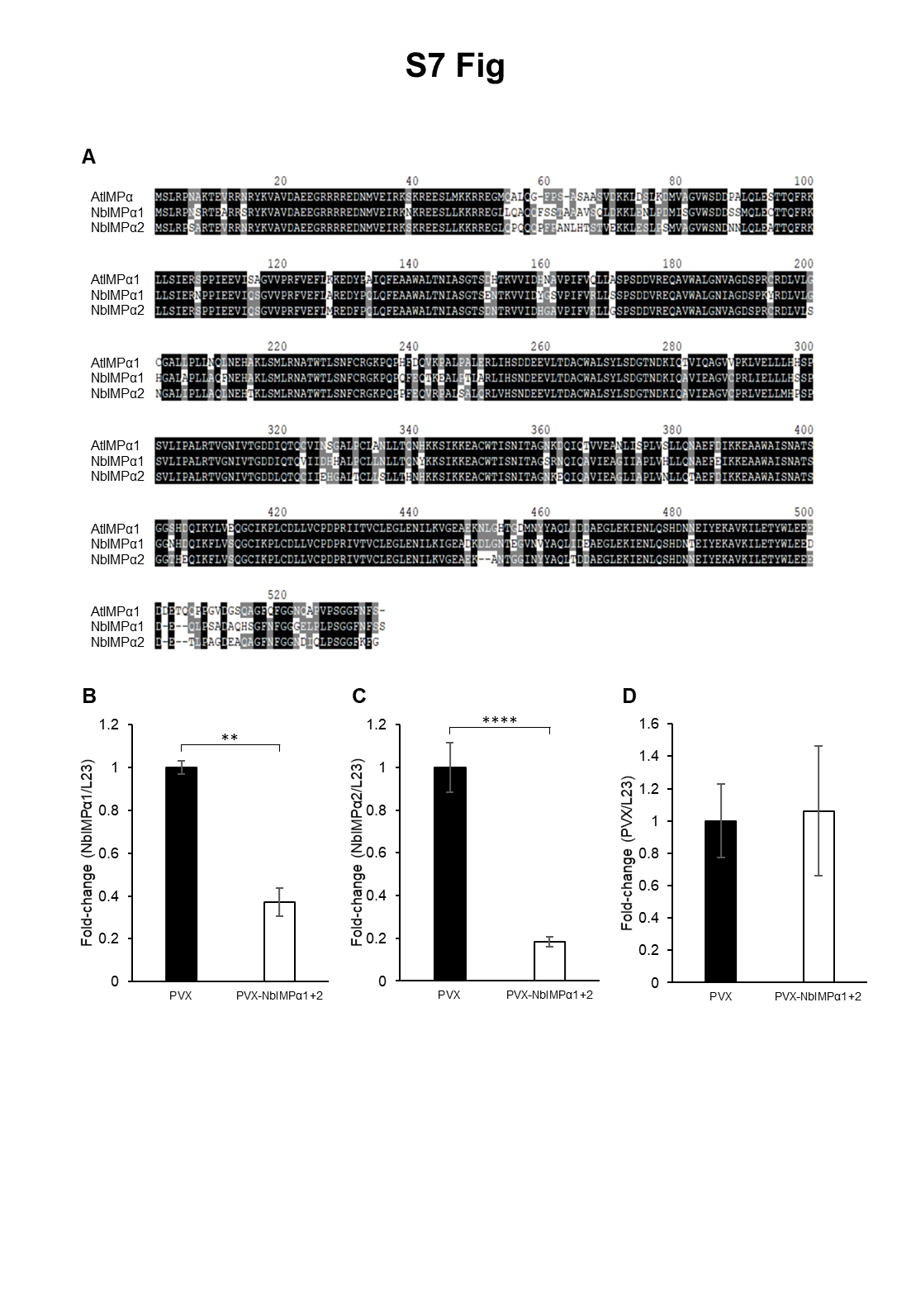

Supplement: S7 Fig — (A) Alignment of AtIMPα1 and two IMP orthologues (NbIMPα1 and NbIMPα2) of N. benthamiana. The amino acid positions are indicated above the alignment. (B and C) Real-time RT-PCR quantification of NbIMPα1 and NbIMPα2 transcripts in the silenced N. benthamiana plants. A partial sequence of either NbIMPα1 or NbIMPα2 was cloned into the PVX vector in a reverse orientation (PVX-IMPα1 or PVX-IMPα2). N. benthamiana plants were co-inoculated with PVX-IMPα1 and PVX-IMPα2 (PVX-IMPα1+2) to simultaneously silence NbIMPα1 and NbIMPα2. Total RNA extracts from the infected leaves were analyzed at 15 dpi. Mean values (±SE) (n = 4) are fold-changes calculated when the value of PVX is set to 1.0, and the values were analyzed on log-transformed data for a significant difference using Student’s t-test (**P < 0.01, ****P < 0.0001). (D) The PVX RNA levels were quantified by real-time RT-PCR. Mean values (±SE) (n = 5) are fold-changes calculated when the value of PVX is set to 1.0, and the values were analyzed on log-transformed data for a significant difference using Student’s t-test (P > 0.05). (TIF) [file ppat.1010267.s007.tif]

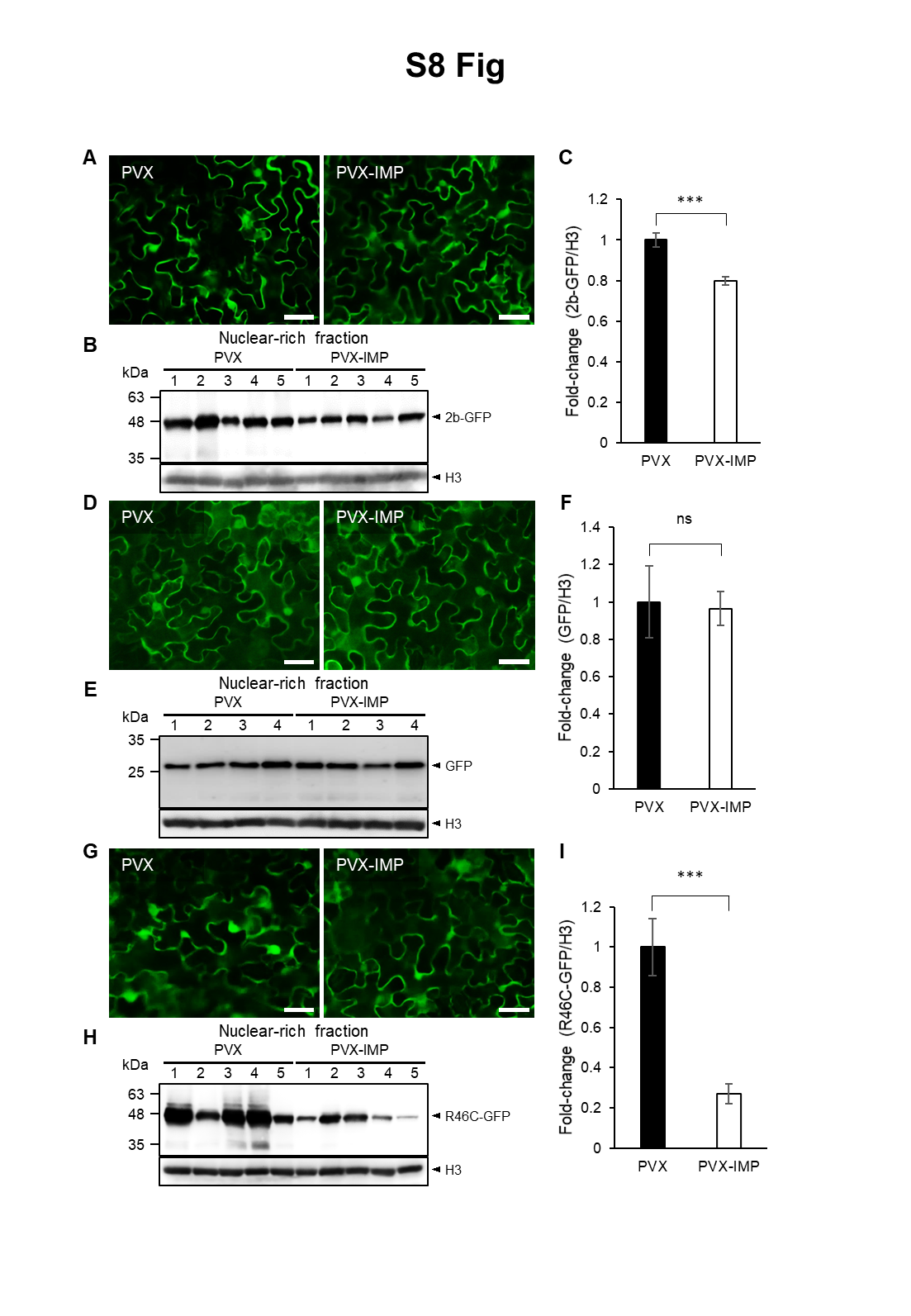

Supplement: S8 Fig — (A–C) 2b-GFP was overexpressed by agroinfiltration in PVX- or PVX-IMP-infected N. benthamiana at 15 dpi. GFP fluorescence was observed at 2 days post-infiltration using a Leica DMI 6000B (A). Scale bar: 50 μm. (B) Western blot of 2b-GFP in the nuclear-rich fraction detected using anti-GFP antibodies. The numbers above the blot are five plants replicates. H3 was detected using anti-H3 antibodies as a loading control. (C) The relative 2b-GFP/H3 ratio was densitometrically calculated using Multi Gauge Software (Fujifilm) and indicated as a fold-change value. Mean values (±SE) were compared on log-transformed data for significant differences using Student’s t-test (***P < 0.001). (D–F) The free GFP was expressed as a control by agroinfiltration in PVX- or PVX-IMP-infected N. benthamiana. (D) Subcellular localization of the GFP control at 2 days post-infiltration. (E) Western blot analysis for GFP and H3 using the nuclear-rich fractions. The numbers above the blot are four plants replicates. (F) Relative GFP/H3 ratio was densitometrically calculated using Multi Gauge Software (Fujifilm) and indicated as a fold-change. Mean values (±SE) were compared on log-transformed data for a significant difference using Student’s t-test. (G–I) Effect of IMPα silencing on the subcellular localization of the 2b-R46C mutant (R46C), which has very weak RSS activity. The R46C-fused GFP construct (R46C-GFP) was overexpressed by agroinfiltration in the PVX- or PVX-IMP-infected N. benthamiana. (G) Subcellular localization of R46C at 2 days post-infiltration. (H) Western blot of R46C-GFP and H3 detected as described above. The numbers above the blot are five plants replicates. (I) Relative 2b-GFP/H3 ratio was densitometrically calculated using the Multi Gauge Software (Fujifilm) and indicated as fold-changes. Mean values (±SE) were compared on log-transformed data for a significant difference using Student’s t-test (***P < 0.01). (TIF) [file ppat.1010267.s008.tif]

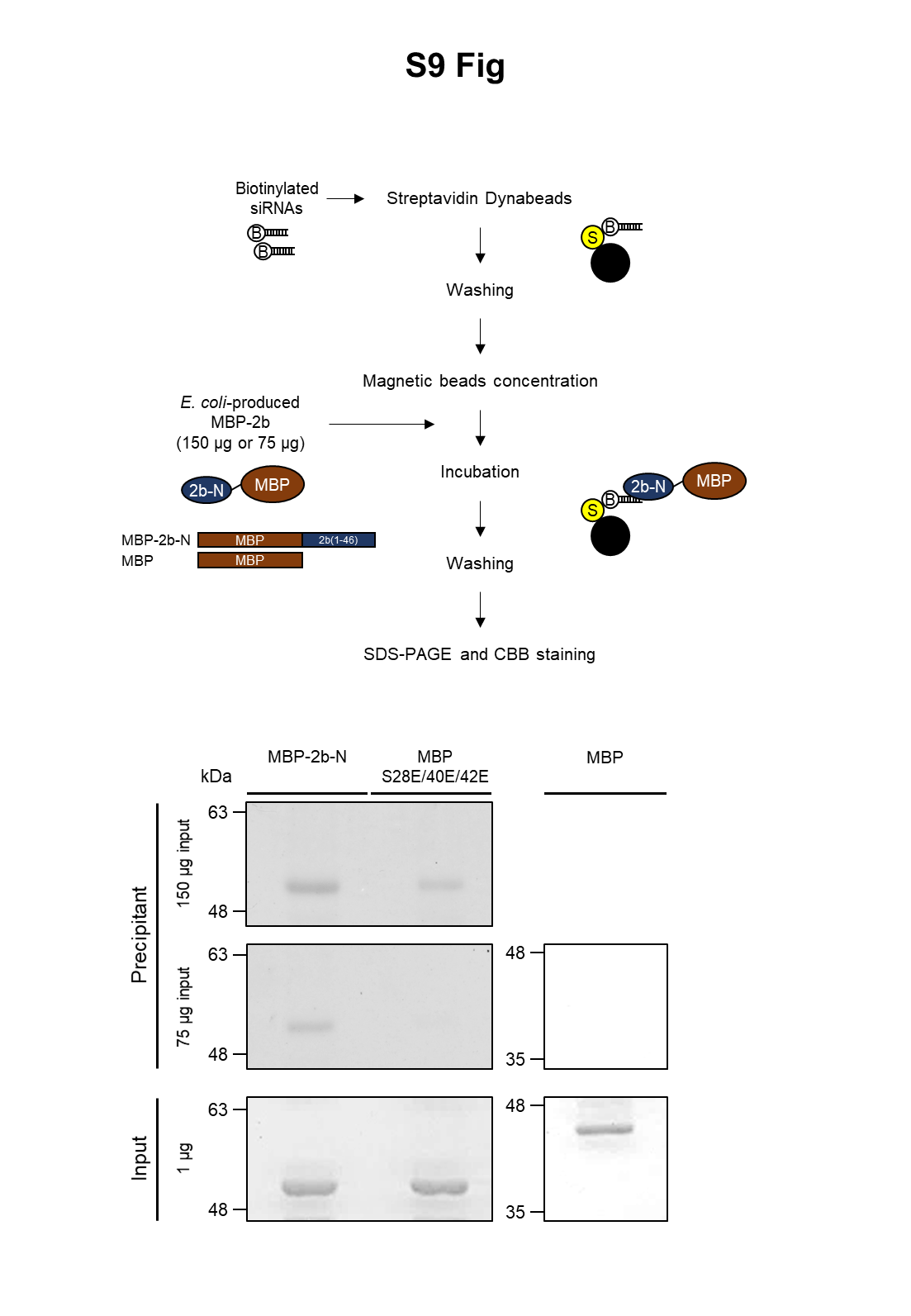

Supplement: S9 Fig — MBP was fused to the N-terminal region (46 amino acid region) of 2b (MBP-2b). The recombinant proteins were synthesized in E. coli and column-purified. The purified proteins were then co-incubated with biotin-labeled 21-nt siRNA (5′-UUGCUCAACAGUAUGGGCAUU-biotin-3′). The recombinant proteins were co-precipitated by the biotin–siRNA interaction using Dynabeads M-280 Streptavidin (Invitrogen) and detected in 10% polyacrylamide gel by CBB staining. MBP was used as a control. (TIF) [file ppat.1010267.s009.tif]

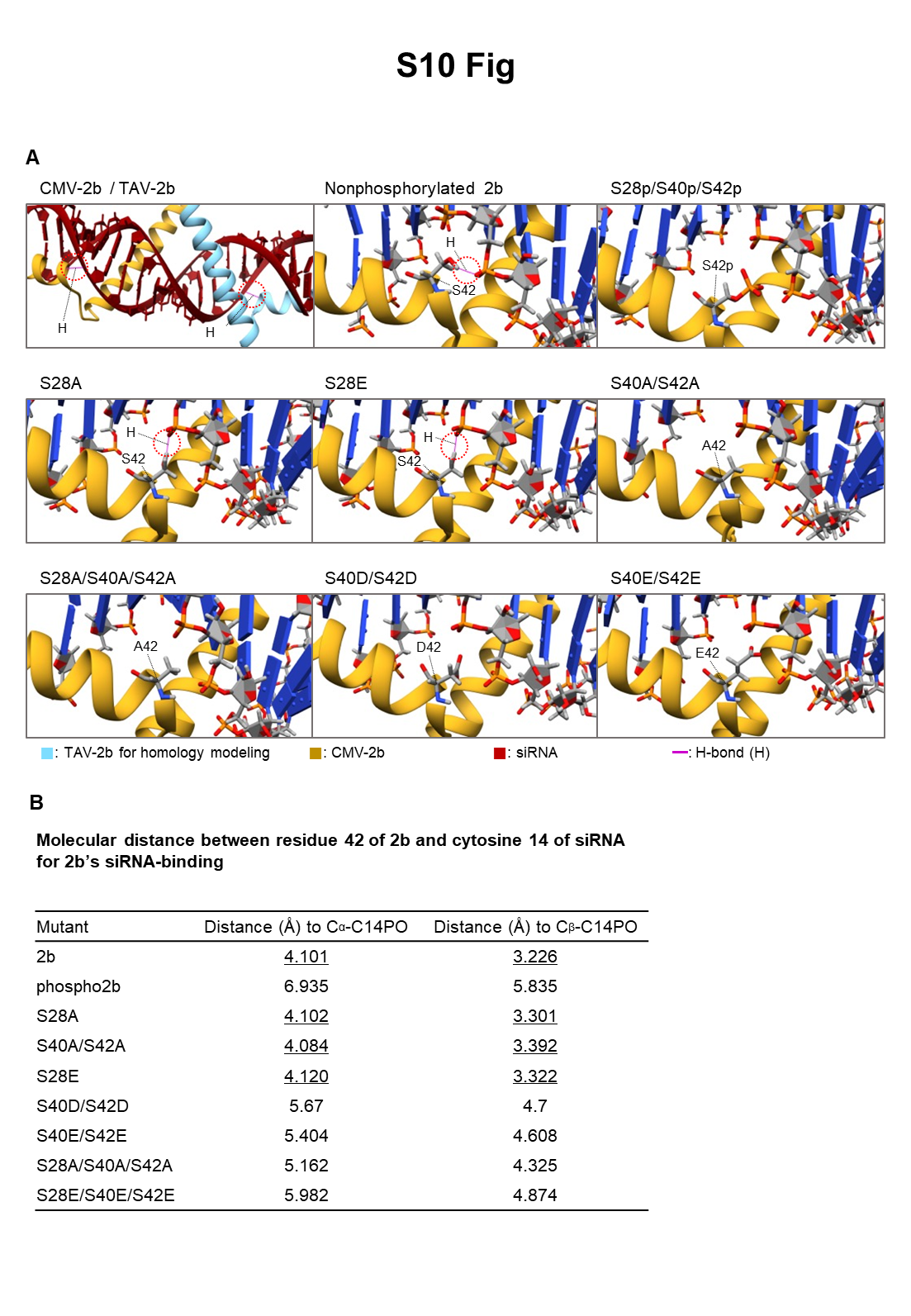

Supplement: S10 Fig — The crystallography of the TAV 2b protein (PDB ID: 2ZI0) of TAV has been reported [49]; data are available in the Research Collaboratory for Structural Bioinformatics Protein Data Bank (RCSB PDB) [80]. The protein structure of TAV 2b was used for homology modeling of CMV 2b. Structures of wild-type CMV 2b and its mutants were modeled by I-TASSER v5.1 (https://zhanglab.ccmb.med.umich.edu/I-TASSER/) [81]. The 2b structures in the state of siRNA binding are representative low-energy conformations after the global torsion was minimized using ICM-Pro. The predicted structures were visualized and matched with TAV 2b using ICM-Pro (Molsoft, San Diego, CA, USA), and those structures are low-energy conformations after the global torsion were minimized in ICM-Pro. H-bond (H) formation (red-dashed circles) was examined using ICM-Pro and UCSF chimera [82] (A). The distances between the alpha/beta carbon (Cα/Cβ) of S42 and the phosphate oxygen of cytosine 14 (C14PO) of siRNA were calculated using UCSF chimera (B). 2bs with some RSS activity (Fig 4) are underlined. The H-bond in S40A/S42A was not created because S42 was replaced with an alanine residue and thus does not have a hydroxyl group for H-bond formation. Note that 2b’s RSS activity seems to be correlated to the distance between the hydroxyl group of the serine residue 42 of 2b and the phosphate oxygen of the cytosine residue 14 of siRNA that are involved in the H-bond. The importance of this H-bond was also suggested by Nemes et al. (2017) [31]. (TIF) [file ppat.1010267.s010.tif]

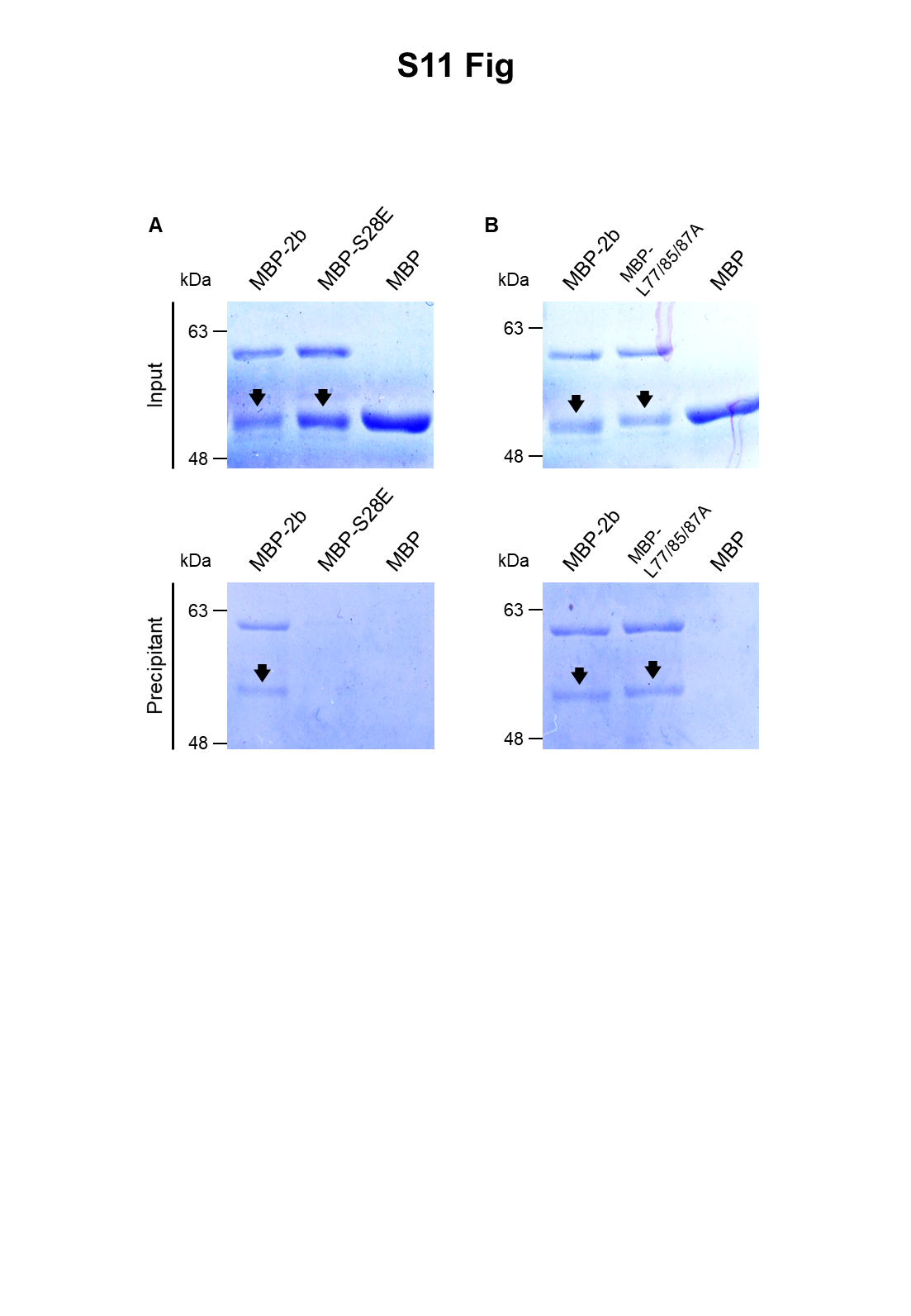

Supplement: S11 Fig — MBP was fused to the N-terminal of 2b, S28E and S77/85/87A. The recombinant proteins were synthesized in E. coli and column-purified. The co-precipitation of biotin-labeled siRNA and the E. coli-synthesized proteins were treated as described in S9 Fig. The amounts of the recombinant proteins were compared between 2b and S28E (A) or between 2b and S77/85/87A (B). The MBP-fused proteins were detected in a 10% polyacrylamide gel by CBB staining. The black arrowheads indicate 2b fused with a truncated form of MBP, which is often generated in E. coli [47]. (TIF) [file ppat.1010267.s011.tif]

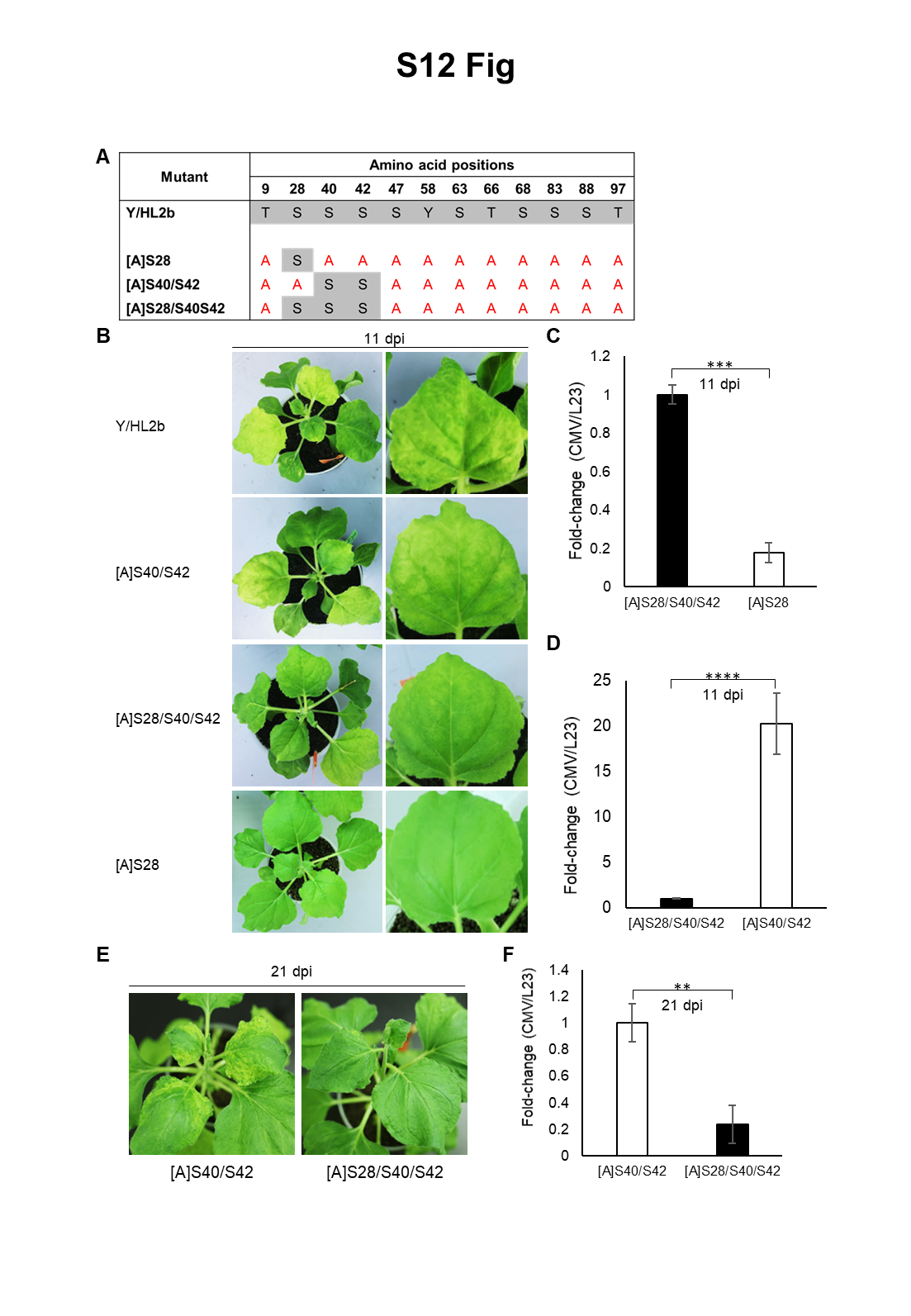

Supplement: S12 Fig — (A) Mutants with alanine substitutions at various putative phosphorylation sites were created, and CMV RNA 2 constructs with those 2b mutants were prepared by inserting the 2b fragments into the CMV H1 vector. (B) Symptom severity on N. benthamiana plants inoculated with the CMV constructs carrying the various 2b mutants. [A]S28, [A]S40/S42 and [A]S28/S40/S42 were created using the primers in S1 Table. The mutant 2b backbone with alanine residues at all the putative phosphorylation sites is shown as “[A]”. Symptoms were observed at 11 dpi. (C, D) Relative accumulation level of CMV RNA 3 in systemically infected upper leaves determined by real-time RT-PCR using primer pairs listed in S1 Table. The values of fold-changes were analyzed on log-transformed data by Student’s t-test (***P < 0.001, ****P < 0.0001). (E) Symptoms on upper leaves of the plants 21 days after inoculation (dpi) with either CMV:[A]S40/S42 or CMV:[A]S28/S40/S42. (F) Relative accumulation levels of CMV RNA 3 and 4 in the plants infected with CMV:[A]S40/S42 or CMV:[A]S28/S40/S42 at 21 dpi. Tissues were collected from the leaves in S9E Fig for quantitative RT-PCR. Means were compared on log-transformed data for a significant difference using Student’s t-test (**P < 0.01). (TIF) [file ppat.1010267.s012.tif]

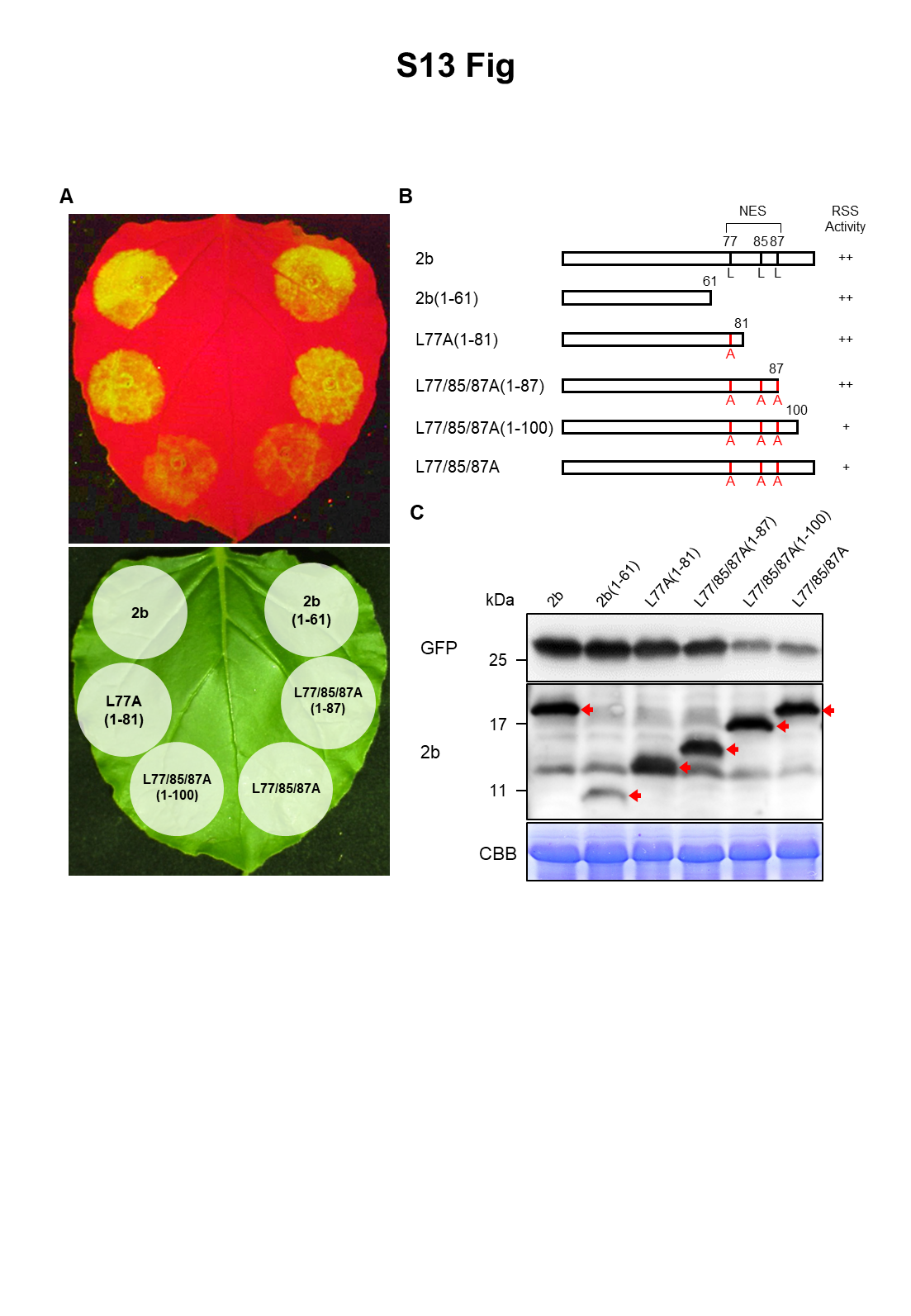

Supplement: S13 Fig — (A) RSS activity of 2b, S77/85/87A and the C-terminal deletion mutants. A series of C-terminal deletion mutants were created using S77/85/87A as the background (B), and their RSS activity was examined as described in Fig 6B. (C) The expression levels of GFP and 2b were estimated by western blot analysis using anti-GFP and anti-2b antibodies, respectively. Red arrows indicate 2b and the 2b mutants. RuBisCo large subunit is shown as a loading control. (TIF) [file ppat.1010267.s013.tif]

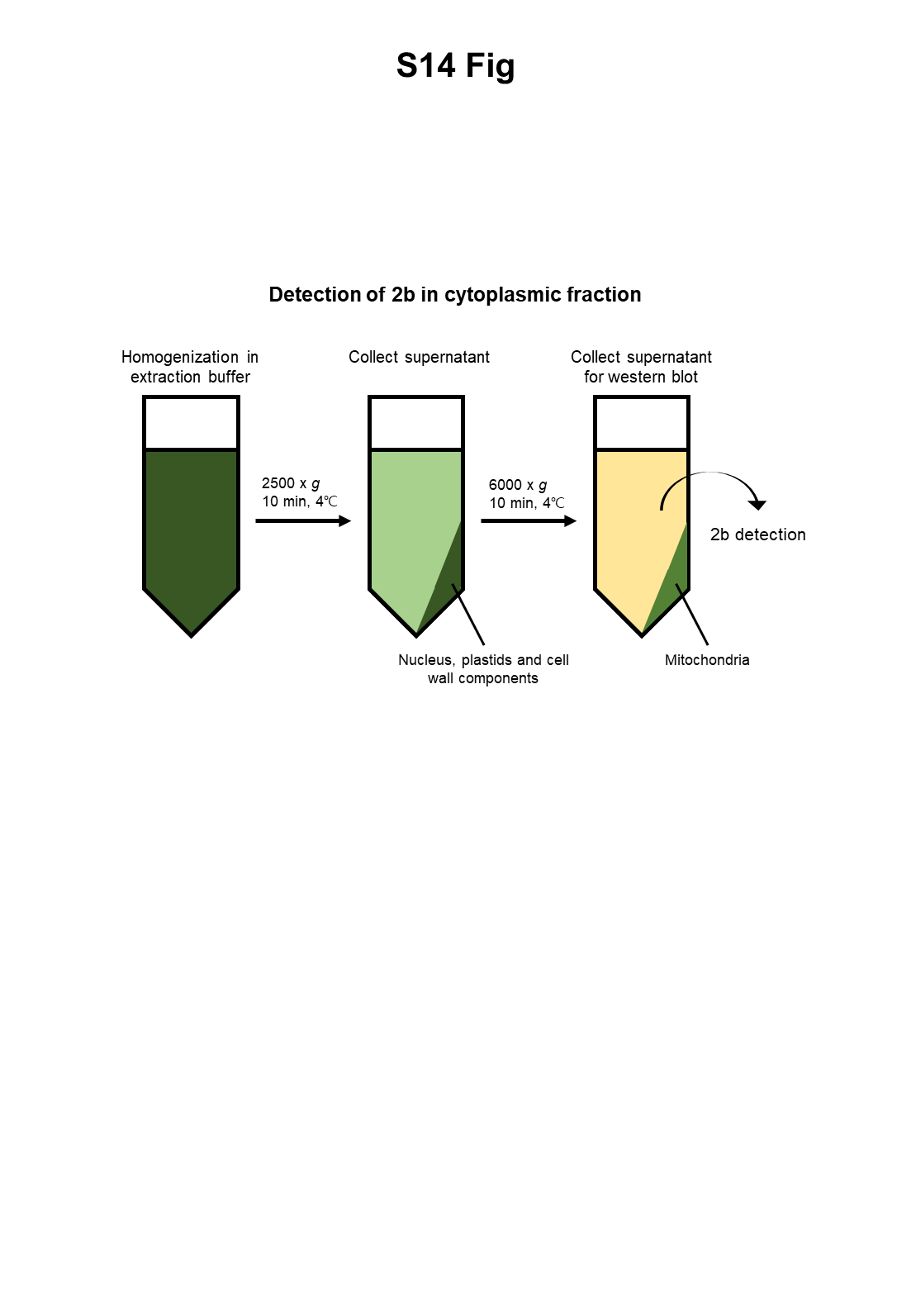

Supplement: S14 Fig — Agroinfiltrated leaf tissues were homogenized in extraction buffer (0.35 M sucrose, 50 mM Tris-Cl, pH 7.5, 5 mM MgCl2, 2 mM 2-mercaptoethanol), and the cytoplasmic fraction was separated by a series of differential centrifugations. The supernatant after the final centrifugation was used for western blot analysis. (TIF) [file ppat.1010267.s014.tif]

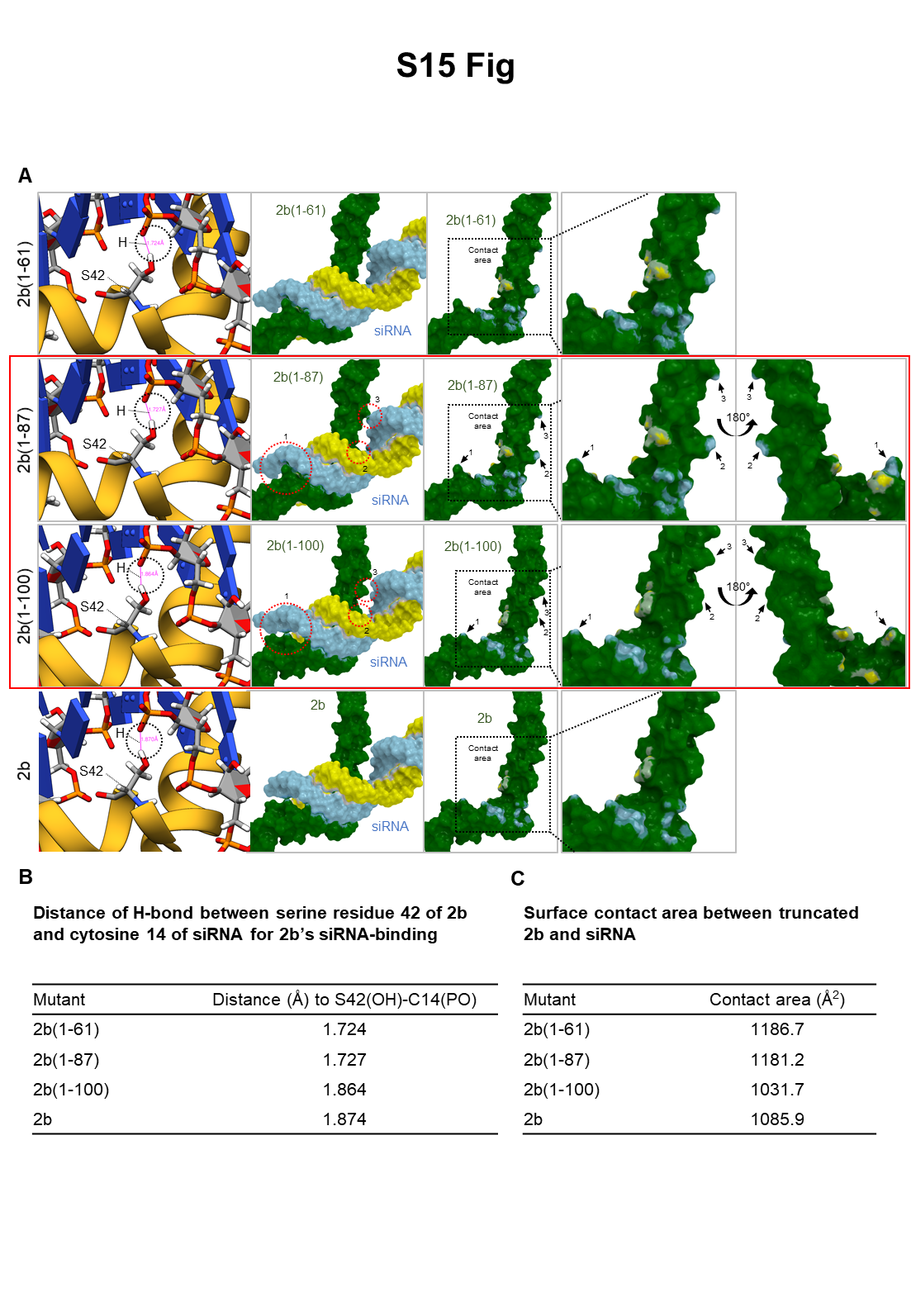

Supplement: S15 Fig — (A) Based on the 2b-siRNA binding model in S10 Fig, the 3D structures of the siRNA-2b complexes using wild-type 2b and the C-terminal deletion mutants were constructed, and the global energy of each complex was optimized by minimizing torsion in ICM-Pro. The H-bond (H) between S42 and cytosine 14 (C14) (black-dotted circles) were visualized using both UCSF Chimera and ICM-Pro programs. The molecular surface areas of 2b and siRNA were generated in ICM-Pro and their surface contact area were analyzed. The regions in the red-dotted circles and black arrows (indicated as 1–3) are the points where the overlapped areas were clearly different between 2b(1–87) and 2b(1–100). (B) The distance of the H-bond between the hydroxyl group of S42 and the phosphate of C14 was measured in the UCSF Chimera. (C) The interface areas shared by 2b and siRNA were calculated using ICM-Pro. (TIF) [file ppat.1010267.s015.tif]

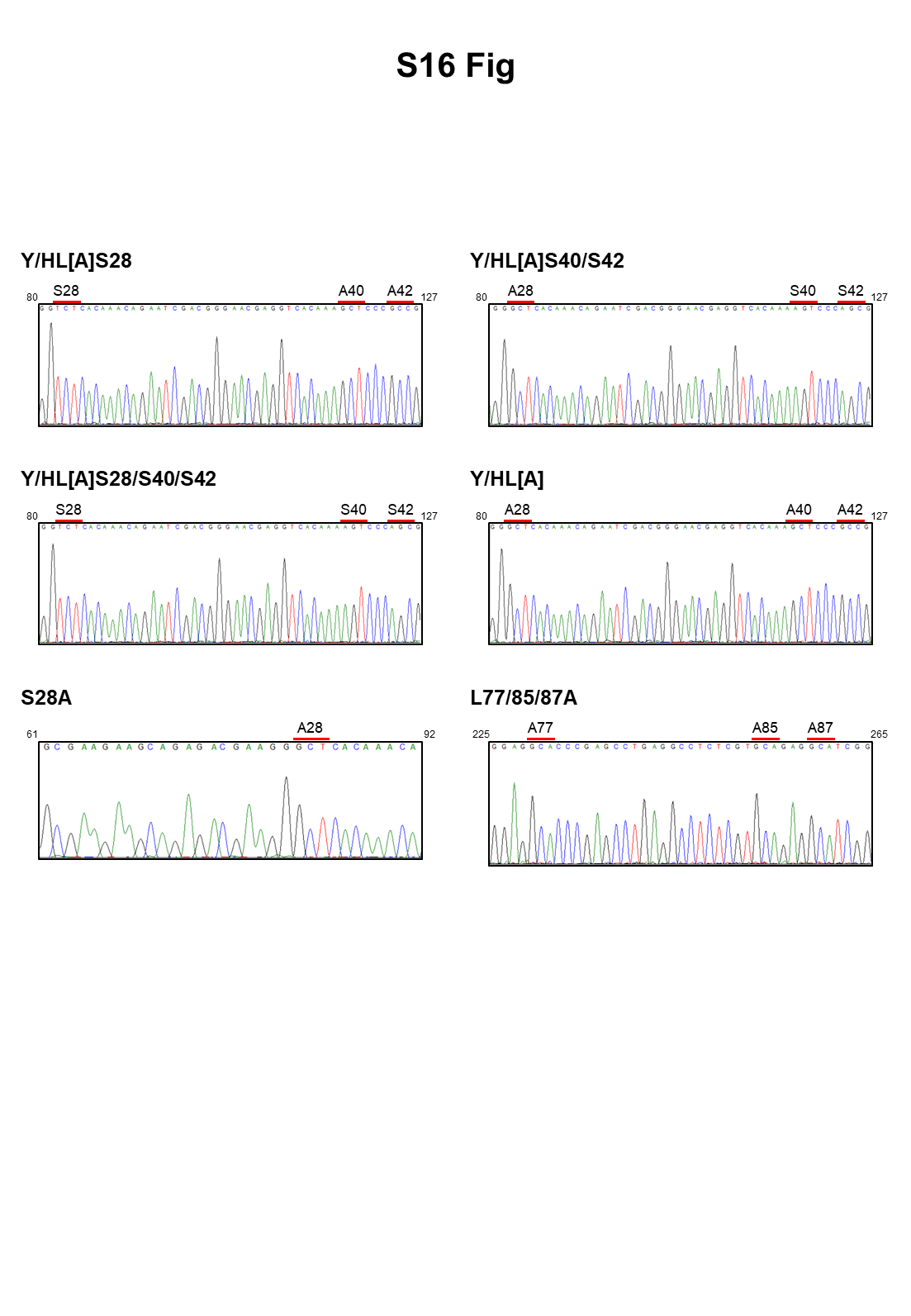

Supplement: S16 Fig — To confirm that the 2b’s mutations are maintained during viral infection, the entire 2b sequences were amplified by RT-PCR from the infected plants at 15 dpi. Their sequences were analyzed by the BigDye terminator method. The wave raw data of the sequencing analyses were shown. Each codon of the mutations is indicated in red bars. (TIF) [file ppat.1010267.s016.tif]
