# Supplementary material for: Importin/exportin-mediated nucleocytoplasmic shuttling of cucumber mosaic virus 2b protein is required for 2b’s efficient suppression of RNA silencing
Source: PLoS Pathog. 2022 Jan 26;18(1):e1010267. doi: 10.1371/journal.ppat.1010267 (PMC8820599; doi:10.1371/journal.ppat.1010267)
Supplement: S1 Table — (PDF) [file ppat.1010267.s017.pdf]

**S1 Table** Primers used for cloning and real-time RT-PCR.

| Primer | Primer sequence (5′–3′) | Mutation | Vector |
|---|---|---|---|
| 2b-5-T=A | ATGGAATTGAACGTAGGTGCAATGGCAAACGTCG | T9A | pMAL-c2x, CMV-H1 |
| 2b-5-S28A | CAGAGACGAAGGGCTCACAAACAG | S28A | pMAL-c2x, CMV-H1, pBE2113 |
| 2b-3-S28A | CTGTTTGTGAGCCCTTCGTCTCTG | | |
| R2b-S28E-5 | AGAGACGAAGGGAACACAAACAGA | S28E | pBE2113 |
| R2b-S28E-3 | TCTGTTTGTGTTCCCTTCGTCTCT | | |
| 2b-5-S=A | GGTCACAAAGCTCCCGCCGAGAGAGCGCG | S40A/S42A | pMAL-c2x, CMV-H1, pBE2113 |
| 2b-3-S=A | CGCGCTCTCTCGGCGGGAGCTTTGTGACC | | |
| 2b-5-A42S | CACAAAGCTCCCAGCGAGAGAG | A42S | pMAL-c2x, CMV-H1 |
| 2b-3-A42S | CTCTCTCGCTGGGAGCTTTGTG | | |
| R2b-S40E-S42E-5 | AGGTCACAAAGAACCCGAAGAGAGAGCG | S40E/S42E | pBE2113 |
| R2b-S40E-S42E-3 | CGCTCTCTCTTCGGGTTCTTTGTGACCT | | |
| R2b-S40D-S42D-5 | AGGTCACAAAGATCCCGACGAGAGAGCG | S40D/S42D | pBE2113 |
| R2b-S40D-S42D-3 | CGCTCTCTCGTCGGGATCTTTGTGACCT | | |
| 2b-5-S45A | AGAGAGCGCGTGCAAATCTCAGA | S45A | CMV-H1 |
| 2b-3-S45A | TCTGAGATTTGCACGCGCTCTCT | | |
| 2b-5-Y58A | CTACCGTTCGCTCAAGTAGATGG | Y58A | CMV-H1 |
| 2b-3-Y58A | CCATCTACTTGAGCGAACGGTAG | | |
| 2b-5-63/66/68A | GGTGCGGAACTGGCAGGGGCATGCCGCCAT | S63A/T66A/S68A | CMV-H1 |
| 2b-3-63/66/68A | GCATGCCCCTGCCAGTTCCGCACCATCTAC | | |
| 2b-5-A63S | CAAGATGGTTCGGAACTGGCAGG | A63S | CMV-H1 |
| 2b-3-A63S | CCTGCCAGTTCCGAACCATCTTG | | |
| 2b-5-A66T | GATGGTGCGGAACTGACAGGGGCATG | A66T | CMV-H1 |
| 2b-3-A66T | CATGCCCCTGTCAGTTCCGCACCAT | | |
| 2b-5-A68S | GAACTGGCAGGGTCATGCCGCCAT | A68S | CMV-H1 |
| 2b-3-A68S | ATGGCGGCATGACCCTGCCAGTTC | | |
| 2b-5-83/88A | TCGTTTAGAGTTAGCGGCGGAAG | S83A/S88A | CMV-H1 |
| 2b-3-83/88A | CGCTAACTCTAAAGGAGCGGCCTC | | |
| 2b-5-A83S | GAGCCTGAGGCCTCTCGTTTAGAG | A83S | CMV-H1 |
| 2b-3-A83S | CTCTAAACGAGAGGCCTCAGGCTC | | |
| 2b-5-A88S | GTTTAGAGTTATCGGCGGAAG | A88S | CMV-H1 |
| 2b-3-A88S | CTTCCGCCGATAACTCTAAAC | | |
| 2b-5-T97A | CTTGACGATGCAGATTGGTTCG | T97A | CMV-H1 |
| 2b-3-T97A | CGAACCAATCTGCATCGTCAAG | | |
| R2b-L77-85-87A-F2 | GAGGCACCCGAGCCTGAGGCCTCTCGTGCAGAGGCATCG | 2b-ΔNES | pBE2113, CMV-H1 |
| R2b-L77-85-87A-R2 | CGATGCCTCTGCACGAGAGGCCTCAGGCTCGGGTGCCTC | | |
| y2b-5-Xb | GCTCTAGAATGGAATTGAACGTAGGTGC | | pBE2113 |
| S65T-3-Sc | CGCGAGCTCATGAAGTGACAGATAGTTATTTGTATAGTTCATCC | | |
| y2b-3-Sc | TCCGAGCTCTCAGAAAGCACCTTCCGC | | |
| y2b-3-Flag-Sc | GAGCTCCTACTTGTCATCGTCGTCCTTGTAGTCGAAAGCACCTTCCGCCCA | | |
| y2b-5-Ec | GCGAATTCATGGAATTGAACGTAGGTG | | pMAL-c2x |
| y2b-3-Sal | GCGGATCCTCAGAAAGCACCTTCCGCCCATTTGTT | | |
| y2b-3-1-138Sal | GTCGACTCAACGCGCTCTCTCAGCGGG | | |
| y2b-S42A-3-138-Sal | GTCGACTCAACGCGCTCTCTCGCTGGG | | |
| y2b-3-1-210Sal | GCGTCGACTCAGCGGCATGACCCTGTCAGTTC | | |
| y2b-5-Stu | TAGAAGGCCTGACGCGTGACTAGTAACCTCCCCTTCCGCATC | | CMV-H1 |
| y2b-3-Spe | GGACTAGTTCAGAAAGCACCTTCCGCCC | | |
| IMPa1-5-Bm | AGGATCCATGTCGCTGAGGCCGAACTCG | | pBE2113 |
| IMPa1-3-Flag-Sc | GAGCTCCTACTTGTCATCGTCGTCCTTGTAGTCTGAACTGAAGTTGAATCCTC | | |
| S65T-5-168 | TCACGGCAGACAAACAAAAG | | |
| S65T-3-168 | AAAGGGCAGATTGTGTGGAC | | |
| CMV-DET-5-340 | GTTGACGTCGAGCACCAACGC | | |
| CMV-DET-3-340 | TGGTCTCCTTTTGGAGGCCC | | |
| Nb-L23-5-110 | AAGGATGCCGTGAAGAAGATGT | | |
| Nb-L23-3-110 | GCATCGTAGTCAGGAGTCAACC | | |
